# Gut microbiota responses to isocaloric macronutrient modulation in tissue-specific insulin resistance: a secondary analysis of the PERSonalized glucose Optimization through Nutritional intervention (PERSON) randomized trial

**DOI:** 10.1016/j.ajcnut.2026.101340

**Published:** 2026-05-05

**Authors:** Kelly M Jardon, Alexander Umanets, Koen Venema, Inez Trouwborst, Anouk Gijbels, Gabby B Hul, Els Siebelink, Lydia A Afman, Gijs H Goossens, Ellen E Blaak

**Affiliations:** 1 TiFN, Wageningen, The Netherlands; 2 Department of Human Biology, NUTRIM - School of Nutrition and Translational Research in Metabolism, Maastricht University Medical Center+, Maastricht, The Netherlands; 3 Centre for Healthy Eating and Food Innovation, Maastricht University Campus Venlo, Venlo, The Netherlands; 4 Division of Human Nutrition and Health, Wageningen University, Wageningen, The Netherlands

**Keywords:** precision nutrition, insulin resistance, gut microbiota, metabolic phenotyping, obesity

## Abstract

**Background:**

Precision nutrition strategies can be effective in optimizing health outcomes. We previously showed that dietary macronutrient modulation targeting tissue-specific insulin resistance (IR) phenotypes induced pronounced improvements in cardiometabolic health. It remains unclear whether these improvements may partially be explained by gut microbiota-related mechanisms.

**Objectives:**

We investigated whether 12-wk high monounsaturated fatty acid (HMUFA) and low-fat, high-protein, high-fiber diets (LFHP) impact gut microbiota composition and functionality in people with predominant muscle IR (MIR) compared with liver IR (LIR) in relation to cardiometabolic health improvements.

**Methods:**

This 2-center, randomized, double-blind, dietary intervention trial included 179 individuals with LIR or MIR [40‒75 y, body mass index (in kg/m^2^) 25‒40], who followed either a 12-wk isocaloric HMUFA or LFHP diet. A 7-point oral glucose tolerance test was performed to determine tissue-specific IR and cardiometabolic risk factors. Fecal microbiota composition was profiled using 16S ribosomal ribonucleic acid amplicon sequencing (V3‒V4 region), and GLP-1 and gut microbial products were determined in plasma and feces.

**Results:**

The HMUFA diet induced significant shifts in overall gut microbial composition (*P* < 0.05) and short-chain fatty acid-producing bacteria (q < 0.05) in the LIR phenotype, but not in MIR. The LFHP diet induced only modest changes in gut microbiota features. We found phenotype-specific correlations between specific baseline taxa abundance and change in metabolic outcomes (MIR-HMUFA: *Barnesiella-*ΔMISI (Spearman ρ = 0.45, *P* < 0.001); LIR-HMUFA: *Sutterella*-Δplasma-C-reactive protein (Spearman ρ = 0.57, *P* = 0.0001) and a Rhodospirillales genus-Δhomeostasis model assessment of insulin resistance (Spearman ρ = ‒0.58, *P* < 0.001).

**Conclusions:**

Individuals with predominant LIR seem more prone to diet-induced gut microbiota-related improvements in cardiometabolic health than those with MIR, highlighting the importance of understanding heterogeneity in IR. Our findings support a role for the gut microbiota in precision nutrition targeting tissue-specific IR.

Clinicaltrials.gov registration: This is a secondary analysis of the PERSonalized glucose Optimization through Nutritional intervention (PERSON) randomized trial. Registration number: NCT03708419, https://clinicaltrials.gov/study/NCT03708419.

## Introduction

Diet is an important modifiable lifestyle factor contributing to cardiometabolic health, and adherence to a healthy dietary pattern may prevent or delay the onset of obesity and related conditions such as insulin resistance (IR), type 2 diabetes, and cardiovascular diseases [1,2]. Despite the progression in our understanding of lifestyle interventions aiming to improve cardiometabolic health, long-term adherence to generic dietary recommendations is poor, and the desired health improvements are suboptimal [3]. Implementing more targeted, precision nutrition approaches based on individual characteristics or metabolic phenotype may improve outcomes of lifestyle interventions [[4], [5], [6]].

We previously reported that modulation of dietary macronutrient composition based on tissue-specific IR phenotypes, either more pronounced liver IR (LIR) or muscle IR (MIR), leads to a pronounced improvement in cardiometabolic health benefits [7]. Specifically, individuals with LIR showed greater improvements in whole-body (Matsuda index) and skeletal muscle insulin sensitivity [muscle insulin sensitivity index (MISI), serum triglycerides (TG), and serum C-reactive protein (CRP)] on a low-fat, high-protein, high-fiber diet (LFHP). Individuals with predominant MIR benefited more from a high MUFA diet (HMUFA). Until now, the underlying mechanisms for the differential dietary intervention response in tissue-specific IR remain unclear.

The gut microbiota has been consistently recognized for its role in human cardiometabolic health and disease, and response to dietary interventions [8,9]. Indeed, baseline gut microbiota composition predicted both acute postprandial glucose and lipid responses in a general population, and longer-term diet-induced cardiometabolic changes in people living with obesity [10,11]. Because modulation of microbial composition by dietary fiber or by fecal transplant experiments showed more pronounced effects on peripheral IR than hepatic IR, alterations in gut microbiome features may thus contribute to the previously found differential effects of dietary intervention on cardiometabolic health outcomes in individuals with distinct tissue-specific IR [12,13].

Importantly, we showed that LIR and MIR exhibit a differential microbial composition and functionality, with the LIR phenotype having a higher abundance of short-chain fatty acid (SCFA)-producing taxa [14]. Similarly, it has previously been shown that the overall gut microbiota composition is altered in individuals with impaired glucose tolerance (characterized among other factors by MIR), whereas microbial composition of individuals with impaired fasting glucose (characterized by LIR) resembles more that of healthy individuals [[15], [16], [17]].

Based on the above findings, we hypothesized that the gut microbiota and related gut metabolites may partially explain the differential diet-induced effects on cardiometabolic health in individuals with distinct tissue-specific IR phenotypes. Here, we performed a secondary analysis using data from the PERSonalized glucose Optimization through Nutritional intervention (PERSON) study [18] to evaluate the effects of the 12-wk LFHP and HMUFA diets on gut microbiota composition and related metabolite production in individuals with LIR or MIR. In addition, we determined the association between microbial species and diet-induced changes in cardiometabolic health parameters.

## Methods

### Study design and participants

The current study is a secondary analysis of the PERSON study, a 2-center, randomized, double-blinded, 12-wk dietary intervention study with a parallel design. The study design and methodology have been described previously [18]. The study was conducted from May 2018 until November 2021 at Maastricht University Medical Center+ (MUMC+) and Wageningen University in the Netherlands, in line with the principles of the Declaration of Helsinki. The protocol was approved by the medical ethical committee of the MUMC+, Maastricht, the Netherlands (NL63768.068.17) and registered at clinicaltrials.gov (NCT03708419). All participants gave written informed consent. Participants were not involved in the design of the study. All participants received a personal report of the study outcomes after study completion. Results will be disseminated to the general public.

Inclusion criteria were as follows: age 40 to 75 y, BMI (in kg/m^2^) 25 to 40, body weight stability for ≥3 mo (no weight gain or loss >3 kg), and tissue-specific IR, characterized as predominant LIR or MIR. Tissue-specific IR was assessed at screening, and the MISI and hepatic IR index (HIRI) were calculated based on the plasma glucose and insulin concentrations during a 7-point oral glucose tolerance test (OGTT) [[18], [19], [20]]. Tertile cutoffs for MISI and HIRI from a previous study with a similar study population [21,22] were used to identify individuals with predominant MIR or LIR. Exclusion criteria included prediagnosis of type 2 diabetes, diseases or use of medication that affect glucose and/or lipid metabolism, major gastrointestinal diseases, history of major abdominal surgery, uncontrolled hypertension, smoking, alcohol consumption >14 units/wk, and >4 h/wk moderate-to-vigorous physical activity. We additionally excluded participants who did not collect fecal samples according to the study protocol or who used antibiotics during the intervention period (*n* = 42). Missing data were excluded and reported in the individual analyses. Eligible participants started the study within 3 mo after screening.

### Study diets and randomization

A total of 242 participants were included and randomly allocated to either PhenoDiet group A or PhenoDiet group B, which consisted of unique combinations of the MIR and LIR metabolic phenotypes and 2 diets, including an HMUFA and an LFHP diet [18]. In the HMUFA diet, key products included olive oil, olives, olive tapenade, and low-fat margarine with olive oil. Key products for the LFHP diet were low-fat yogurt and quark, reduced-fat cheese, very low-fat spread, pumpkin seeds, baking margarine with olive oil, and a dietary fiber supplement (2 g β-glucan per 6 g, DSM Nutritional Products) providing 6 to 12 g of additional fiber per day.

PhenoDiet group A included individuals with MIR following the HMUFA diet and individuals with LIR following the LFHP diet. PhenoDiet group B included individuals with LIR and MIR on HMUFA and LFHP diets, respectively. Random allocation to either PhenoDiet group A or PhenoDiet group B in a 1:1 ratio was conducted by an independent researcher using center-specific minimization, with randomization factors of 1.0 for the LIR/MIR phenotype, and 0.8 for age and sex, and a base probability of 0.7 by means of biased coin [23]. Both researchers and participants were blinded to the participants’ metabolic phenotype (LIR or MIR), and thus blinded to the phenotype × diet combination. In the present study, we focused our analysis on diet effects (HMUFA, LFHP) within the phenotypes LIR and MIR (phenotype × diet interactions).

The HMUFA diet had a targeted macronutrient composition of 38% of energy from fat (20% MUFA, 8% PUFA, 8% SFA), 48% of energy from carbohydrates (CHO) (30% polysaccharides; 3 g/MJ fiber), and 14% of energy from protein. The macronutrient composition of the LFHP diet was targeted at 28% of energy from fat (10% MUFA, 8% PUFA, 8% SFA), 48% of energy from CHO (30% polysaccharides; >4 g/MJ fiber), and 24% of energy from protein (Table 1). Energy from CHO was similar between diets. Key food products were provided in prequantified amounts. The LFHP diet included a dietary fiber supplement (2 g oat β-glucan per 6 g product, DSM-Firmenich) providing 6‒12 g of additional fiber per day. Alcohol consumption was restricted to ≤1 glass per day. Both diets were in agreement with the Dutch dietary guidelines [24]. Participants received verbal and written dietary instructions at the start of the intervention and received weekly dietary counseling. Body weight was monitored every week to ensure participants remained relatively weight-stable throughout the intervention. The details of the dietary intervention are described elsewhere [18].

**TABLE 1:** Targeted nutrient composition of the high MUFA diet and *low-fat, high-protein, high-fiber diet* intervention diets

	HMUFA	LFHP
Fat (En%)	38	28
Monounsaturated fat	20	10
Polyunsaturated fat	8	8
Saturated fat	8	8
Protein (En%)	14	24
Animal-based, % of total protein	45	60
Plant-based, % of total protein	55	40
Carbohydrates (En%)	48	48
Mono- and disaccharides	12	12
Polysaccharides	30	30
Fiber, g/MJ	3	>4
Alcohol, g/d	<3	<3

Abbreviations: En%, energy percentage from total daily intake; HMUFA, high monounsaturated fatty acid diet; LFHP, low-fat, high-protein, high-fiber diet.

### Sample size calculation

The sample size in the PERSON study was based on expected differences in effects between PhenoDiet groups A and B on the original study’s primary outcome: the disposition index [7]. We previously estimated the required sample size to detect a standardized effect size of 0.46 with a power of 90% to be *n* = 202; *n* = 240 in total to allow for a 15% dropout. We did not perform a power calculation for this secondary analysis.

### Assessment of dietary adherence

Dietary compliance was assessed by 3 unannounced 1-d weighted food records on 2 nonconsecutive weekdays and 1 weekend day using the mobile application “Traqq” [25]. In addition, plasma fatty acid profile was measured by nuclear magnetic resonance spectroscopy as a biomarker for MUFA, PUFA, and SFA consumption [26]. Dietary compliance in this study population has been reported previously [7].

### Habitual dietary intake

A validated 163-item semiquantitative food frequency questionnaire was used to assess habitual dietary intake prior to the start of the dietary intervention period [27]. Dietary misreporting was evaluated by Goldberg’s method, using the ratio of daily energy intake (EI) to estimated basal metabolic rate (BMR). Energy under- (EI/BMR < 0.87) and over-reporters (EI/BMR > 2.75) were excluded from data analyses. Here, a total of *n* = 22 was excluded due to underreporting and *n* = 1 due to overreporting.

### Clinical measurements

#### Anthropometrics and blood pressure

Body weight (kg) and height (cm), and waist and hip circumference (cm) were measured to the closest 0.1 unit in duplicate and in underwear. Systolic and diastolic blood pressure were measured in triplicate on the nondominant arm with an automated sphygmomanometer after a 5-min rest. The first measurement was used to acclimatize the participant to the measurements and was therefore excluded from the data.

#### 7-point OGTT

Whole-body and tissue-specific (liver and muscle) insulin sensitivity were estimated based on a 7-point OGTT at baseline and after 12 wk of intervention. Participants ingested a 200 mL, 75-g glucose solution (Novolab) after an overnight fast. Blood draws were performed from the antecubital vein at t = 0 min, t = 15 min, t = 30 min, t = 45 min, t = 60 min, t = 90 min, and t = 120 min, and plasma glucose and insulin were determined. Fasting samples were used to determine serum TG and plasma CRP concentrations [7]. Glucose and insulin values were used to calculate the HOMA-IR and Matsuda index for whole-body insulin sensitivity, HIRI for liver insulin sensitivity, and the MISI for muscle insulin sensitivity, as described previously [18]. The Matsuda index [28], HIRI, and MISI [19] have been validated against the gold standard hyperinsulinemic clamp technique. All blood biochemical assays were previously described in detail [7].

### Plasma analyses

#### Plasma glucagon-like peptide 1

Plasma glucagon-like peptide 1 (GLP-1) was determined in fasted condition (t = 0) and at t = 30, t = 60, t = 90, t = 120, and t = 180 after ingesting a liquid high-fat mixed meal [total 350 g containing 2.8 MJ, 49 g (64 energy percentage, en%) fat, 48 g (29 en%) CHO, 12 g (7 en%) protein] [18]. We calculated the AUC using the trapezoid method for the postprandial GLP-1 response. For analysis, 40 *μ*L of dipeptidyl peptidase-IV inhibitor (Merck) was added to EDTA and Aprotinin (Becton, Dickinson and Company) tubes, respectively. Plasma samples were assayed for total GLP-1 immunoreactivity using an antiserum that reacts equally with intact GLP-1 and the primary (N-terminally truncated) metabolite as previously described [29]. Analyses were performed in a subgroup of the study cohort (*n* = 124).

#### Circulating SCFA and branched-chain fatty acid

A fasting blood sample was collected in a 4 mL Lithium Heparin tube (Becton, Dickinson and Company) for the measurement of plasma SCFA and branched-chain fatty acid (BCFA) concentrations using liquid chromatography–mass spectrometry. The liquid chromatography was performed using a micro flow ultra-high-performance liquid chromatography instrument (Dionex UltiMate 3000) using protocols as described previously [30,31]. Analyses were performed in a subgroup of the study cohort (MUMC+ study center, *n* = 79).

### Fecal analyses

#### Fecal sample collection

Fecal samples were collected at home and stored for a maximum of 24 h in the participants’ freezer at ‒20°C. Samples were kept frozen during transport from collection to storage. After arrival in the laboratory, the samples were stored at ‒80°C prior to further analysis.

#### Microbiota analysis

Sequencing of the V3-V4 region of the 16S rRNA gene was performed to determine microbiota composition as reported before [32,33]. In short, the QIAamp fast DNA stool mini kits (Qiagen) were used for genomic DNA isolation. Barcoded amplicons from the V3-V4 region of 16S rRNA genes were generated using a 2-step PCR. In the first step, 10 to 25 ng genomic DNA was used as template for the first PCR with a total volume of 50 *μ*L using the 341F (5’-CCTACGGGNGGCWGCAG-3’) and 785R (5’-GACTACHVGGGTATCTAATCC-3’) primers appended with Illumina adaptor sequences. PCR products were purified (QIAquick PCR purification kit), the size of the PCR products was checked on a fragment analyzer (Advanced Analytical), and concentration was quantified by fluorometric analysis (Qubit dsDNA High Sensitivity (HS) assay kit). Purified PCR products were used for the second PCR in combination with sample-specific barcoded primers (Nextera XT index kit, Illumina). Subsequently, PCR products were purified, checked on a fragment analyzer, and quantified, followed by equimolar multiplexing, and sequencing on an Illumina MiSeq with the paired-end (2x) 300 bp protocol (Illumina). The sequencing data were analysed and demultiplexed using the standard Illumina pipeline based on sample specific barcodes, yielding FASTQ files in the CASAVA 1.8 format. Quantitative Insights Into Microbial Ecology 2 software was used for initial microbial analyses [34]. Reads were imported as Quantitative Insights Into Microbial Ecology 2 artifacts, and the dada2 plug-in was used for denosing and construction of amplicon sequence variant (ASV) table [35]. The taxonomic assignment of ASVs was performed with a Naïve Bayes classifier trained on full-length 16S rRNA gene sequences from the SILVA v138 database [36]. A phylogenetic tree of the ASVs’ representative sequences was inferred from MAFFT (Multiple Alignment using Fast Fourier Transform) sequence alignment using the FastTree approach. Samples with low total reads number (<5000 reads) were removed from further analysis.

#### Fecal SCFA and BCFA

Fecal samples were used to measure fecal SCFA and BCFA using ion exchange chromatography with conductivity detection (Brightlabs). Fecal matter was centrifuged at 14,000 x *g;*10 min, 4°C, filtered through a 0.45 *μ*m polytetrafluoroethylene (PTFE) filter, and diluted in the mobile phase (1.5 mM aqueous sulfuric acid). Ten microliters were loaded into the column with the help of an automatic sampler 730 (Metrohm). The acids were eluted according to their acid dissociation (pKa) values. The analysis was carried out by ion exclusion chromatography using an 883 IC chromatograph (Metrohm) equipped with a Transgenomic ICSep ICE-ION-300 column (30 cm × 7.8 mm × 7 *μ*m) and a MetroSep RP2 Guard. A column flow of 0.4 mL/min with a column temperature of 65°C was used. The acids were detected using suppressed conductivity detection. Analyses were performed in a subgroup of the study cohort (MUMC+ center, *n* = 87).

### Statistics

#### Clinical parameters

Differential phenotype-specific intervention effects of the HMUFA and LFHP diets on cardiometabolic parameters and circulating and fecal metabolites were assessed by analysis of covariance with the model (wk 12 ∼ wk 0 + diet + phenotype + dietxphenotype + center + age + sex). Skewed variables were log-transformed (log_10_) to improve normality. Two-tailed false discovery rate adjusted q < 0.05 was considered statistically significant. Analyses were performed using IBM SPSS Statistics (version 28).

#### Gut microbiota

Downstream microbiota and metabolite-related analyses were performed in R v4.1.2 programming language and environment [37]. Using the “qiime2R” package [38], the ASV table, rooted phylogenetic tree, taxonomic table, and supporting data were imported and combined into a phyloseq object [39]. Prior to further analysis, ASVs with fewer than 10 reads across all samples, not assigned to any bacterial phylum, and assigned to chloroplasts or mitochondria were removed. Taxonomic names were shortened to the last assigned taxonomic level. Duplicated names were made unique by adding a level identifier with a count number (e.g., g1, g2, … g*n*). Statistical analyses were stratified for diet groups (HMUFA and LFHP) and diet × phenotype groups (HMUFA-MIR, HMUFA-LIR, LFHP-MIR, LFHP-LIR). Only paired samples (pre- and postintervention) were included.

For the estimation of α diversity, 2 different approaches were used. First, Shannon index, inverse Simpson index (phyloseq), phylogenic diversity (picante) [40], and number of observed taxa were calculated as measures of α-diversity. ASV count per sample was normalized by rarefaction (phyloseq) prior to calculation. Diet effects on α diversity were assessed by the Wilcoxon rank sum test with continuity correction (“stats” package) [37]. Second, Shannon and Simpson indices were calculated at the genus level without prior count normalization as implemented in the “DivNet” package [41,42], followed by statistical testing with linear mixed models (LMM) as implemented in the breakaway package “betta_random” [41] to assess the effect of time with participant ID as a random effect. For both approaches, *P* < 0.05 was considered statistically significant.

Diet-induced shifts in overall microbial composition were investigated by calculating dissimilarity distances between samples at the ASV taxonomic level, followed by testing the strength and significance of grouping with permutational multivariate analysis of variance (ADONIS) as implemented in the “vegan” package (“adonis2”) [43]. Prior to dissimilarity distance calculations, the ASV count was normalized with cumulative sum scaling as implemented in the “metagenomeSeq” package [44]. Dissimilarity distances (weighted and unweighted UniFrac, Jaccard, Bray-Curtis) were calculated using the “phyloseq” package. The following model, *(distances) ∼ ParticipantID + Time,* with 9999 permutations blocked by participant ID, was used for ADONIS analysis [43]. This model allowed us to access intraindividual changes induced by diet and partial out between-individual variations. Differences in dispersion before and after intervention were assessed using betadisper and permutest functions from the “vegan” package with a permutation scheme identical to that described for ADONIS analysis. Shifts in the overall bacterial composition were visualized by plotting the first 2D of distance-based redundancy analysis (package “vegan”) as a scatter-plot.

Diet-induced changes in individual taxa abundance in diet and diet-phenotype subgroups were assessed using the “MaAsLin2” package [45]. Differential abundance was tested at the genus taxonomic level, and only taxa with 50% or more prevalence in any diet-phenotype group were used in the analysis. Additionally, a lower prevalence cutoff of 25% was used. After prevalence filtering, data were stratified per group-level, abundance was normalized using TMM (trimmed mean of M-values) [46], and zero-inflated negative binomial regression analysis was built per taxa with time as the response variable. To account for person-specific variations, participant ID was included as the random variable in the statistical model. The resulting *P* values were adjusted using the built-in Benjamini-Hochberg false discovery rate adjustment method. The q-values ≤0.05 were considered to be statistically significant, and ≤0.1 were considered clinically relevant.

Correlations between changes in cardiometabolic outcome parameters and both baseline and changes in microbial taxa at the genus taxonomic level were assessed using Spearman correlation analyses (“stats” package). We included the Matsuda index, MISI, HOMA-IR, plasma triacylglycerol (TAG), and CRP as cardiometabolic outcomes based on previously reported diet-induced changes [7]. Only correlations with a coefficient ≥0.4 and *P* ≤ 0.001 were considered to be relevant. Data processing, visualization, and reporting were performed with “tidyverse” [47], “ggsignif” [48], “broom” [49], and “knitr” [50] R packages.

## Results

### Baseline characteristics

In total, 221 participants completed the PERSON study [7]. After exclusion of people who used antibiotics during the 12-wk intervention period, individuals who were not able to collect a fecal sample at either 1 or both time points, or collected samples of insufficient quality, paired data of 179 participants were included for the present secondary analysis (Supplementary Figure 1). Participant characteristics were similar between the diet-phenotype groups (Table 2). Overall and by definition, the LIR phenotype tended to have a higher fasting glucose value, whereas 2-h glucose and insulin values were higher in the MIR phenotype. Habitual intake of mono- and disaccharides was higher in the LFHP diet group (Supplementary Table 1). We observed that people with the LIR phenotype had a higher total EI compared to people with MIR, which was not significantly different anymore after adjustment for BMR.

**TABLE 2:** Baseline characteristics of participants stratified on tissue-specific insulin resistance phenotypes within the intervention diets

	HMUFA diet		LFHP diet	
	LIR	MIR	LIR	MIR
General characteristics	*n* = 39	*n* = 52	*n* = 38	*n* = 50
Age, y	59.0 ± 8.0	60.5 ± 8.2	59.6 ± 6.6	61.2 ± 8.2
Females, *n* (%)	17 (44.7)	30 (57.7)	17 (44.7)	34 (68.0)
Systolic blood pressure, mmHg	129 ± 16	124 ± 14	124 ± 15	127 ± 13
Diastolic blood pressure, mmHg	80 ± 11	77 ± 11	78 ± 11	79 ± 9
Anthropometrics				
BMI, kg/m^2^	30.9 ± 4.0	29.9 ± 3.5	30.1 ± 3.6	29.4 ± 3.3
Waist circumference, cm	104.7 ± 11.9	101.7 ± 8.6	103.9 ± 9.2	101.4 ± 9.2
Waist-to-hip ratio	0.94 ± 0.10	0.93 ± 0.09	0.96 ± 0.08	0.93 ± 0.09
Glucose metabolism				
HbA1c (mmol/mol)	36.1 (35.3, 36.7)	36.3 (35.5, 37.0)	36.0 (35.5, 36.5)	36.1 (35.6, 36.6)
Fasting glucose, mmol/L	5.4 (5.0, 6.0)	5.3 (5.1, 5.6)	5.4 (4.9, 5.7)	5.2 (5.0, 5.5)
2-h glucose, mmol/L	5.7 (4.8, 7.5)	6.4 (5.4, 7.4)	5.6 (4.6, 6.7)	6.9 (5.6, 7.8)
Fasting insulin, pmol/L	56.7 (41.0, 76.2)	50.3 (39.5, 63.6)	48.1 (37.2, 63.9)	49.9 (37.8, 59.0)
2-h insulin, pmol/L	349.8 (232.6, 637.8)	397.7 (270.6, 509.8)	278.7 (177.2, 490.6)	476.4 (261.3, 715.3)
HOMA-IR, AU	2.1 (1.3, 2.8)	1.7 (1.3, 2.3)	2.1 (1.2, 2.2)	1.7 (1.2, 2.1)
Medication use, *n* (%)				
Antidepressants	4 (10.3)	1 (1.9)	3 (7.9)	5 (10.0)
Antihypertensives	3 (7.7)	12 (23.1)	7 (18.4)	10 (20.0)
Anti-inflammatory	1 (2.6)	8 (15.4)	3 (7.9)	6 (12.0)
Statins	2 (5.1)	5 (9.6)	3 (7.9)	5 (10.0)
Other	11 (28.2)	21 (40.4)	11 (28.9)	16 (32)

Clinical characterization of the LIR and MIR phenotypes, indicated per diet group. Data are shown as mean ± SD for normally distributed data, median (25th percentile, 75th percentile), or *n* (percent) for categorical data.

Abbreviations: HbA1c, hemoglobin A1c; HMUFA, high monounsaturated fatty acid diet; HOMA-IR, homeostasis model assessment of insulin resistance; LFHP, low-fat, high-protein, high-fiber diet; LIR, liver insulin resistance; MIR, muscle insulin resistance; SD, standard deviation.

### Dietary macronutrient composition and cardiometabolic health

We previously reported that improvements in whole-body (Matsuda index and HOMA-IR) and tissue-specific IR (MISI), serum TG, and plasma CRP were most pronounced in PhenoDiet group B, including clusters of people with an LIR phenotype on the HMUFA diet and people with a MIR phenotype on a LFHP (*n* = 199), independent of changes in body weight and body fat percentage [7]. We identified similar phenotype-specific diet effects in the current secondary analysis (*n* = 179) (Supplementary Table 2).

### The metabolic phenotype determines dietary effects on overall gut microbiota composition and diversity

**FIGURE 1:**
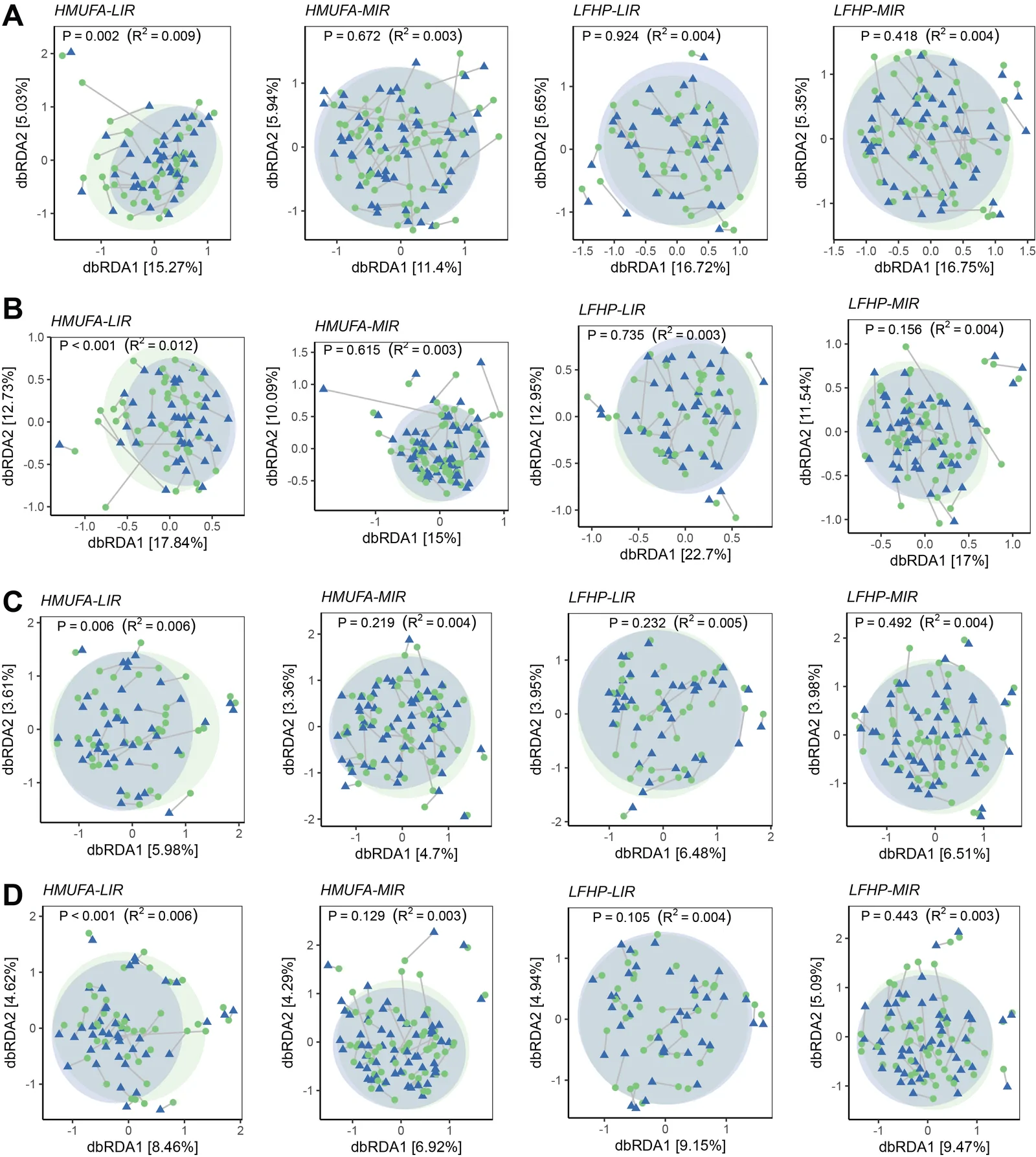
Effects of 12-wk HMUFA and LFHP diets on gut microbiota community structure in people with predominant LIR or MIR. dbRDA plots using unweighted (A) and weighted (B) UniFrac, Jaccard (C), and Bray-Curtis (D) distances in LIR and MIR phenotypes following a diet high in HMUFA or a diet LFHP. *ADONIS* model: distances ∼ participant identification number (ID) + time with 9999 permutation blocked by participant ID. The colors and shapes indicate the phenotypes before and after the 12-wk intervention. The lines indicate diet-induced intraindividual change. dbRDA, distance-based redundancy analysis; HMUFA, high monounsaturated fatty acid diet; LFHP, low-fat, high-protein, high-fiber diet; LIR, liver insulin resistance; MIR, muscle insulin resistance.

We investigated whether the HMUFA and LFHP affected microbial diversity and overall gut microbiota composition in individuals with LIR or MIR. The HMUFA diet induced an overall shift in gut microbiota composition (ADONIS; Bray-Curtis, Jaccard, weighted UniFrac, unweighted UniFrac, all *P* < 0.05) (Supplementary Figure 2, Supplementary Table 3). This shift was dependent on tissue-specific IR phenotype and found in people with LIR but not in MIR (ADONIS; Bray-Curtis, Jaccard, weighted UniFrac, unweighted UniFrac, all *P* < 0.05) (Figure 1, Supplementary Table 4). Although the HMUFA diet-induced shift was significant in the LIR phenotype, the variation in gut microbiota composition was mainly explained by inter-individual differences (R^2^ = 0.78‒0.87), with only a minor contribution of the diet (R^2^ < 0.01). The LFHP diet did not affect overall gut microbiota composition in the total group or the separate LIR and MIR phenotypes.

**FIGURE 2:**
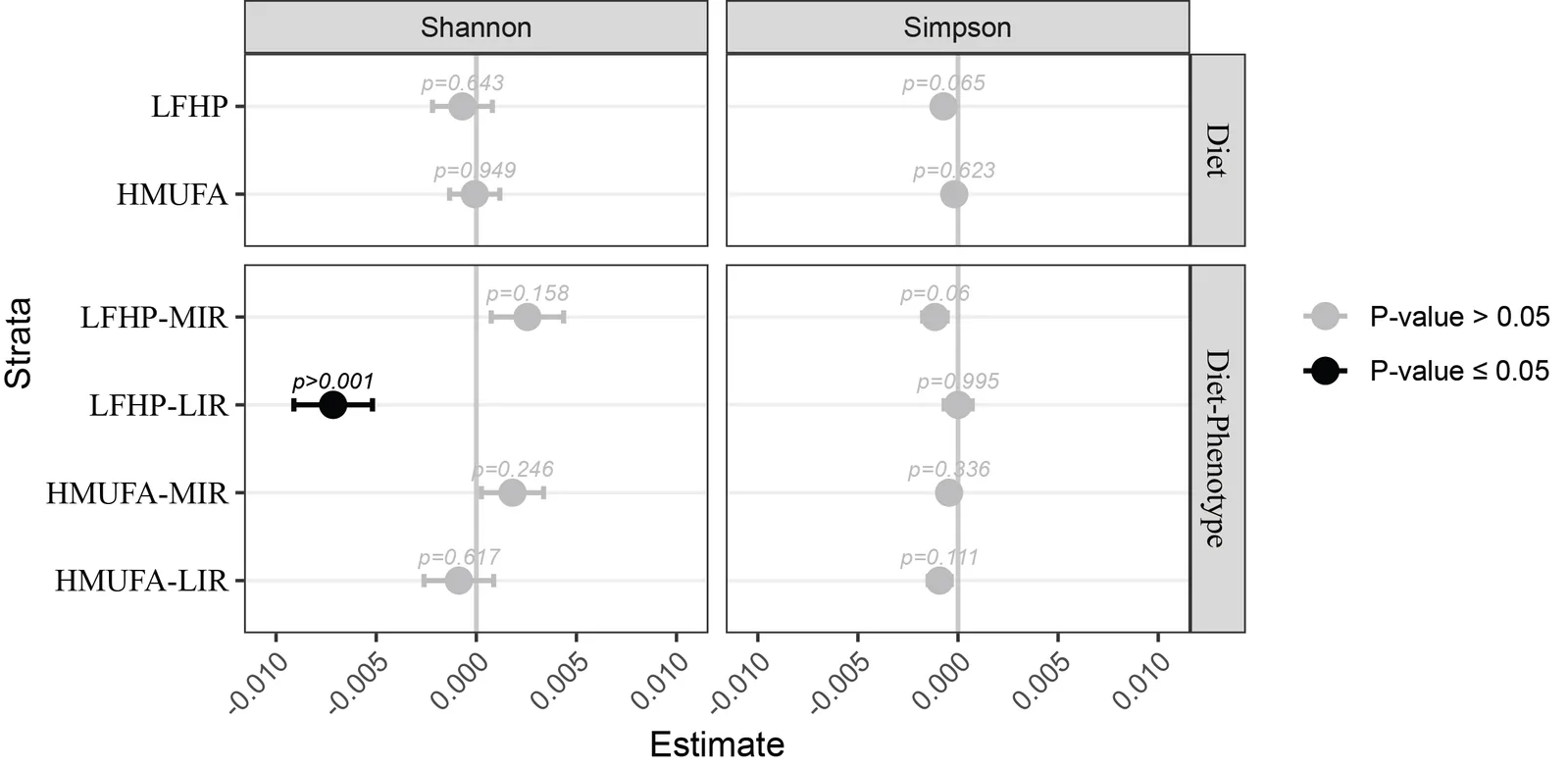
Effects of 12-wk HMUFA and LFHP diets on gut microbiota diversity in people with predominant LIR or MIR. Microbial diversity as assessed using inverted Simpson (InvSimpson) and Shannon indexes (Shannon) for people with MIR or LIR following either a HMUFA or LFHP diet respectively. DivNet - linear mixed model (LMM) model *∼* time with participant identification number (ID) as random effect. *P* < 0.05 for statistical significance. HMUFA, high monounsaturated fatty acid diet; LFHP, low-fat, high-protein, high-fiber diet; LIR, liver insulin resistance; MIR, muscle insulin resistance.

Both HMUFA and LFHP diets did not have pronounced effects on the number of observed taxa, Shannon index, inverse Simpson index, and phylogenic diversity in the total group or the separate LIR and MIR phenotypes (Figure 2, Supplementary Figures 3 and 4, Supplementary Table 5). At the genus level, using the DivNet method, the LFHP diet decreased microbial diversity in the LIR phenotype (Shannon index, Figure 2, Supplementary Table 6).

### Phenotype-specific patterns in diet-induced changes of individual microbial taxa

**FIGURE 3:**
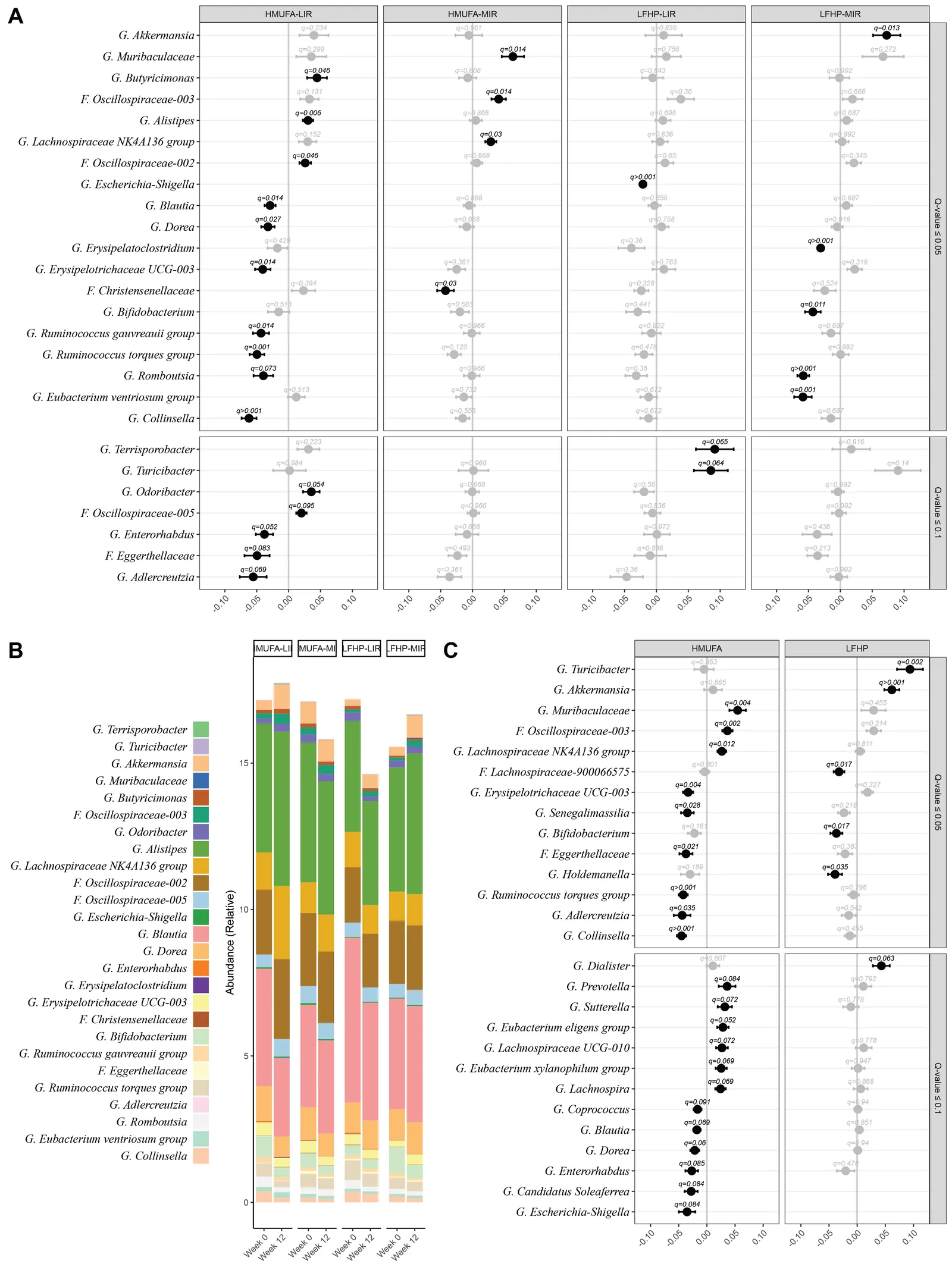
Effects of a 12-wk HMUFA and LFHP diet on individual gut microbial taxa at the genus level. The figure depicts genera significantly (q ≤ 0.1) affected by intervention within diet-phenotype (A and B) or diet (C) groups. (A and C) show regression coefficients +/‒ SE (x-axis) and q-values corresponding to differentially abundant taxa (y-axis) identified by zero-inflated negative binomial regression; color of the dots and error bars indicate if q-value was below (black), or above (gray) set threshold; if the model failed to estimate *P* value, no regression coefficient was plotted. (B) shows cumulative median relative abundance of significantly different taxa across a diet-phenotype group; median relative abundance of individual taxa shown by color within a bar as indicated by legend. The taxa prefix corresponds to the last assign taxonomic level of the genus. ∗q ≤ 0.05. *F*., family; *G*., genus; HMUFA, high monounsaturated fatty acid diet; LFHP, low-fat, high-protein, high-fiber diet; LIR, liver insulin resistance; MIR, muscle insulin resistance; SE, standard error.

Overall, the HMUFA diet induced more pronounced shifts in individual microbial taxa abundance compared to the LFHP diet (Figure 3C, Supplementary Table 7). This pattern was most pronounced in people with the LIR phenotype who followed the HMUFA diet. In this specific phenotype × diet combination (LIR × MUFA), we observed an increase in the genus in the *Oscillospiraceae family, Butyricimonas* and *Alistipes,* and a decrease in *Blautia, Dorea, Erysipelatoclostridium, Ruminococcus gauvreauii group, Ruminococcus torques group, Romboutsia,* and *Collinsella* (Figure 3A, Supplementary Table 7). Of the affected genera in the LIR phenotype on the HMUFA diet, 3 showed a relative abundance of 1% or higher (Figure 3B), and shifted taxa cumulatively covered ∼19.4% of the total gut microbial community at baseline (Supplementary Table 7).

Overall, the LFHP diet induced an increase in the genera *Akkermansia and Turicibacter* (Figure 3C, Supplementary Table 8). The relative abundance of *Bifidobacterium*, a genus in the *Lachnospiraceae* family, and *Holdemanella* decreased. We did observe individual microbial shifts in the LIR and MIR phenotypes, which were unique for the phenotypes on the LFHP diet, but less pronounced compared to the HMUFA-LIR group (Figure 3A, Supplementary Table 7). Overall, findings remain similar when decreasing the prevalence cutoff from >50% to >25% (Supplementary Figure 5, Supplementary Tables 9 and 10).

### Diet-induced effects on plasma GLP-1 and fecal and plasma SCFA and BCFA

At baseline, fecal SCFA and BCFA, as well as postprandial GLP-1, were higher in the LIR (*n* = 89) as compared to the MIR phenotype (*n* = 144), as previously reported [14]. Neither the HMUFA nor the LFHP diets induced major differential shifts in fasting and postprandial plasma GLP-1 and plasma and fecal SCFA and BCFA, as indicators of gut microbiota activity (Table 3).

**TABLE 3:** Effects of the low-fat, high-protein, high-fiber diet and high monounsaturated fatty acid diet on plasma glucagon-like peptide-1 and plasma and fecal short-chain fatty acid and branched-chain fatty acid in liver insulin resistance and muscle insulin resistance

	HMUFA				LFHP				q-value
	LIR		MIR		LIR		MIR		
	Week 0	Week 12	Week 0	Week 12	Week 0	Week 12	Week 0	Week 12	
Plasma									
*GLP-1 (n = 60)*									
Fasting GLP-1, pmol/L	3.5 ± 0.3	4.0 ± 0.4	2.8 ± 0.2	3.0 ± 0.2	3.8 ± 0.3	3.8 ± 0.3	3.6 ± 0.3	3.4 ± 0.2	0.989
Postprandial GLP-1, AUC	2589.7 ± 170.8	2577.1 ± 143.7	2058.2 ± 105.8	2230.8 ± 156.1	2654.5 ± 175.1	2606.6 ± 198.1	2345.2 ± 120.0	2363.8 ± 129.5	0.567
*SCFA (n = 41)*									
Acetate, μmol/L	34.2 ± 4.2	33.8 ± 7.2	32.1 ± 4.1	22.4 ± 2.2	26.1 ± 3.6	23.2 ± 3.0	29.6 ± 2.2	25.5 ± 1.7	0.176
Butyrate, μmol/L	0.3 ± 0.1	0.3 ± 0.1	0.2 ± 0.0	0.3 ± 0.0	0.2 ± 0.0	0.2 ± 0.0	0.3 ± 0.1	0.3 ± 0.1	0.497
Propionate, μmol/L	1.4 ± 0.1	1.4 ± 0.1	1.3 ± 0.1	1.2 ± 0.1	1.6 ± 0.3	1.4 ± 0.2	1.4 ± 0.1	1.5 ± 0.1	0.683
Valerate, μmol/L	2.9 ± 0.2	3.0 ± 0.3	2.6 ± 0,1	2.7 ± 0.2	3.3 ± 0.8	2.8 ± 0.3	2.6 ± 0.3	2.5 ± 0.2	0.49
Hexanoate, μmol/L	0.4 ± 0.0	0.4 ± 0.1	0.4 ± 0.1	0.3 ± 0.0	0.5 ± 0.1	0.4 ± 0.0	0.4 ± 0.0	0.3 ± 0.0	0.678
*BCFA (n = 41)*									
2-methyl butyrate, μmol/L	0.4 ± 0.0	0.4 ± 0.1	0.4 ± 0.1	0.4 ± 0.0	0.4 ± 0.1	0.4 ± 0.1	0.4 ± 0.0	0.4 ± 0.1	0.616
Iso-butyrate, μmol/L	0.4 ± 0.0	0.4 ± 0.1	0.4 ± 0.0	0.3 ± 0.0	0.4 ± 0.1	0.4 ± 0.0	0.3 ± 0.0	0.3 ± 0.0	0.185
Iso-valerate, μmol/L	0.4 ± 0.1	0.4 ± 0.1	0.4 ± 0.0	0.3 ± 0.0	0.4 ± 0.1	0.4 ± 0.1	0.3 ± 0.0	0.3 ± 0.0	0.78
Feces									
*SCFA (n = 50)*									
Acetate, μmol/g	52.2 ± 4.6	55.6 ± 6.1	32.1 ± 2.7	42.5 ± 5.5	54.4 ± 4.7	66.0 ± 9.0	44.1 ± 6.3	38.6 ± 3.1	0.188
Butyrate, μmol/g	16.6 ± 1.4	16.5 ± 1.5	12.5 ± 1.0	15.1 ± 1.5	17.4 ± 1.2	19.1 ± 1.8	16.8 ± 2.4	16.0 ± 1.8	0.675
Propionate, μmol/g	20.4 ± 1.8	19.4 ± 2.1	12.9 ± 1.2	17.0 ± 2.6	21.0 ± 1.7	24.5 ± 3.0	19.3 ± 3.3	18.8 ± 2.5	0.233
*BCFA (n = 50)*									
Iso-butyrate, μmol/g	3.8 ± 0.4	3.7 ± 0.4	2.8 ± 0.3	3.4 ± 0.4	4.0 ± 0.4	4.0 ± 0.4	2.6 ± 0.5	3.3 ± 0.4	0.278
Iso-valerate, μmol/g	3.3 ± 0.3	2.8 ± 0.2	2.4 ± 0.2	2.7 ± 0.3	3.2 ± 0.2	2.9 ± 0.3	2.9 ± 0.3	2.8 ± 0.3	0.578

Data are presented as mean ± SEM and analyzed by ANCOVA adjusted for age, sex, and study center (study center only for GLP-1). *P* values represent group differences with the 12-wk change as the outcome. An adjusted q <0.05 was considered significant.

Abbreviations: ANCOVA, analysis of covariance; AUC, area under the curve; BCFA, branched-chain fatty acid; GLP-1, glucagon-like peptide-1; HMUFA, high monounsaturated fatty acid; LFHP, low-fat, high-protein, high-fiber diet; LIR, liver insulin resistance; MIR, muscle insulin resistance; SCFA, short-chain fatty acid; SEM, standard error of the mean.

### Phenotype-specific associations between the gut microbiota and cardiometabolic parameters

**FIGURE 4:**
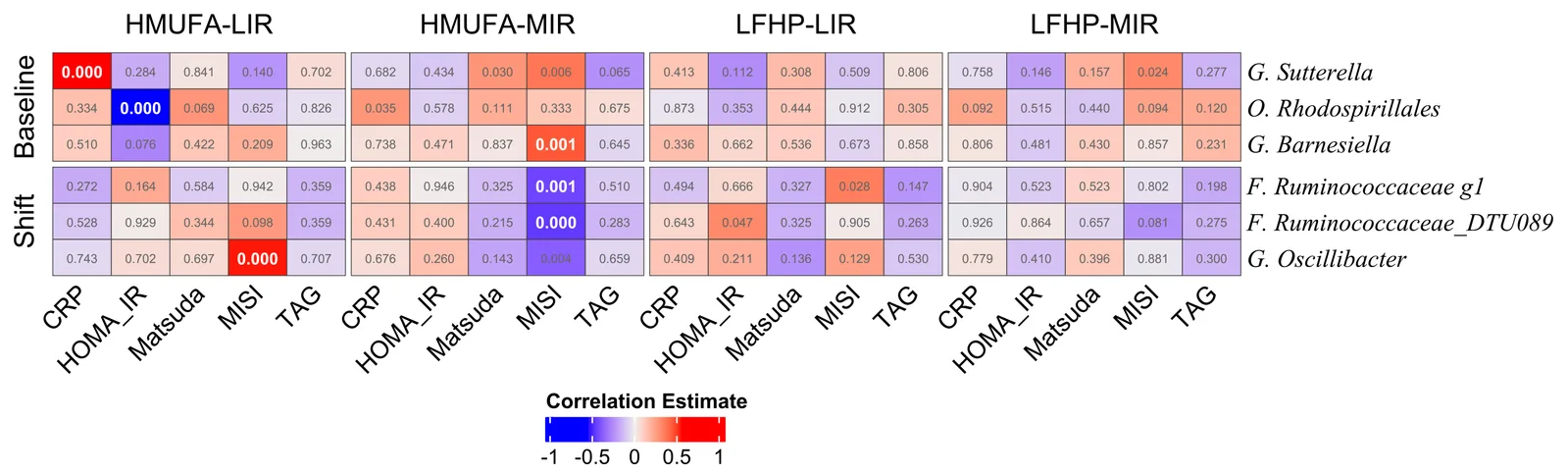
Spearman correlation between selected cardiometabolic outcome parameters and microbial genera abundance. The heatmap visualizes correlations considered important (*P* values ≤0.001 and coefficients ≥0.4). Color hue and intensity represent the direction (positive or negative) and strength of the Spearman correlation, as indicated in the accompanying legend. Numerical values within each cell denote the unadjusted *P* values from the Spearman correlation test. *P* values ≤0.001 are highlighted in white to emphasize important correlations. Cardiometabolic parameters are displayed along the x-axis, and genera along the y-axis. The top annotation indicates the diet-phenotype strata within which correlation analysis was performed. The left annotation specifies whether the analysis was based on genus abundance values at baseline or the change between week 12 and baseline (shift). CRP, C-reactive protein; *F*., family; *G*., genus; HMUFA, high monounsaturated fatty acid diet; HOMA-IR, homeostasis model assessment of insulin resistance; LIR, liver insulin resistance; MIR, muscle insulin resistance; MISI, muscle insulin sensitivity index; O., order; TAG, triacylglycerol.

Next, we assessed whether changes in cardiometabolic outcome parameters (Matsuda index, HOMA-IR, MISI, fasting serum TG, and CRP) following the HMUFA and LFHP diet are associated with either gut microbiota features at baseline or diet-induced shifts in gut microbiota (Figure 4, Supplementary Figure 6, Supplementary Table 11). Microbial composition showed distinct correlations with metabolic outcomes in LIR and MIR.

Baseline relative abundance of the *Barnesiella* genus positively correlated with diet-induced changes in MISI in the HMUFA group in people with the MIR phenotype, but not in the LIR phenotype (Figure 4, Spearman ρ = 0.45, *P* < 0.001). Within the LIR phenotype on the HMUFA diet, we found a positive correlation between baseline *Sutterella* abundance and diet-induced changes in plasma CRP (Spearman ρ = 0.57, *P* = 0.0001), and a negative correlation between the baseline relative abundance of a genus in the Rhodospirillales order and changes in HOMA-IR (Spearman ρ = ‒0.58, *P* < 0.001).

Lastly, we observed associations between changes in MISI and shifts in the abundance of several bacteria. In particular, a positive association with *Oscillibacter* (Spearman ρ = 0.55, *P* < 0.001) in the LIR phenotype following the HMUFA diet, and a negative association with 2 genera from family *Ruminococcaceae* (Spearman ρ = ‒0.44, *P* < 0.001) and family *Ruminococcaceae*-DTU089 (Spearman ρ = ‒0.44, *P* < 0.001) in the MIR phenotype following the HMUFA diet.

## Discussion

We previously established that targeted, tissue-specific IR-based modulation of dietary macronutrient composition enhanced cardiometabolic health improvements [7]. We reported more pronounced improvements of whole-body IR and MIR, fasting serum TG, and plasma CRP for people with LIR on a HMUFA diet, and for people with MIR on an LFHP diet. In the current study, we evaluated for the first time whether features of the gut microbiota may underlie these differential responses to diet in tissue-specific IR. We found that the effects of a 12-wk HMUFA diet on the gut microbiota were dependent on an individual’s tissue-specific IR phenotype. People with the LIR phenotype on the HMUFA diet showed a marginal but significant change in overall microbiota community structure, which was related to shifts in bacteria with SCFA-producing potential representing 19.4% of the total microbial community at baseline. The LFHP diet did not induce changes in overall microbiota composition and had less pronounced effects on individual taxa. Interestingly, correlations between baseline microbial composition and changes therein in MISI, HOMA-IR, and CRP were moderate but distinct in the MIR and LIR phenotype [51]. Our data suggest that the effects of dietary intervention on microbial composition and host metabolism depend on IR phenotype.

On the HMUFA diet, changes in overall microbial community structure and individual taxa abundance were specific for LIR. Interestingly, the HMUFA diet also resulted in the most pronounced cardiometabolic benefits in the LIR phenotype [7], suggesting that these effects may be modulated (to some extent) by changes in microbial composition. HMUFA-induced gut microbiota alterations included both an increase and a decrease in the relative abundance of bacteria with saccharolytic fermentation capacity in LIR. Four of these taxa were the relatively high-abundant bacteria (>1%) *Alistipes*, and an *Oscillospiraceae* genus (both increased with fold changes of respectively 1.28 and 1.27 after intervention), and *Blautia* and *Dorea* (both decreased, fold change 0.72 and 0.65). Decreased *Alistipes* and increased *Blautia* have been previously associated with obesity [52]. Interestingly, *Blautia* has also been inversely associated with visceral adipose tissue mass [53]. Despite these contradictions, the current HMUFA-induced changes confirm a link between these bacteria and an individual’s metabolism, which may be explored further.

Interestingly, we previously reported that people with LIR were characterized by gut microbiota features that were more favorable compared to MIR, including higher abundances of taxa with SCFA-producing potential, and higher fecal SCFA and postprandial GLP-1 responses [14], in line with previous findings in individuals with prediabetes [9]. Having a less dysbiotic and more resilient microbiota may increase the susceptibility to diet-induced microbial changes related to cardiometabolic health outcomes, which may partially explain the HMUFA-induced changes in the LIR phenotype [54]. The MUFA-rich diet resulted in shifts in relative abundances of bacteria with the potency to produce SCFAs in the LIR phenotype, which was not reflected in alterations in gut-related metabolites in both plasma and feces. Of note, plasma and fecal concentrations are a result of net production, absorption, and extraction by the gut and liver, and may have also changed upon intervention.

The LFHP diet only had minor effects on gut microbiota features, but did increase the relative abundance of *Turicibacter* and *Akkermansia*. In general, *Turicibacter* has been recognized for its role in human lipid and bile acid metabolism [55]. However, due to their low relative abundance in this cohort (both <0.01%), the expected physiological effect would be only minimal. *Akkermansia* is widely recognized for having beneficial cardiometabolic health effects [56].

Moreover, only in the HMUFA diet, we identified baseline taxa and shifts in taxa that may predict MISI, HOMA-IR, or CRP values, indicating that dietary intervention success may be predicted to some extent by overall microbial composition within MIR and LIR. In line, baseline characteristics of the gut microbiota are important predictors of acute glycemic or postprandial response after food intake [4,5] and longer-term dietary interventions [10]. Our data expand these findings by showing that the effectiveness of a dietary intervention depends on both metabolic and microbial characteristics of an individual.

The overall lack of LFHP-induced effects on bacteria with anaerobic fermentation capacity is inconsistent with previous studies, in particular dietary interventions including a dietary fiber component [[57], [58], [59]]. As mentioned above, this may relate to the phenotype of individuals and large heterogeneity in responses. Additionally, in our whole-diet approach, fiber intake was increased by consuming both whole-wheat food alternatives, e.g., bread, pasta, and rice, and by the consumption of an additional 6‒12 g of supplemented oat β-glucan. Although oat β-glucan has been shown to improve glucose metabolism, which is in line with our findings, its effects on specific microbial taxa are less consistent [60,61]. An explanation for the lack of LFHP diet-induced changes may be that the beneficial effects of increased fiber intake on saccharolytic fermentation were potentially diminished by the high-protein content (25 E%) of this particular diet, which is supported by the observation that both plasma and fecal SCFA concentrations were largely unaltered in the LFHP group. High-protein diets and proteolytic fermentation products, including ammonia and indoles, have been linked to less beneficial health effects [62]. Proteolytic fermentation may have been increased in the LFHP, not leading to the typical upregulation of saccharolytic fermentation capacity, which is normally accompanying with dietary fiber interventions. Strategies to optimize saccharolytic fermentation capacity within a whole-diet approach may subsequently further improve cardiometabolic health effects and should therefore be investigated further. The HMUFA diet induced changes, however, extend previous findings that consumption of a MUFA-rich diet has previously shown to increase gut microbiota diversity and the abundance of beneficial SCFA-producing bacteria *Prevotella*, *Faecalibacterium prausnitzii,* and *Bifidobacterium* in people living with obesity and shows that these shifts may be phenotype specific [63].

A strength of this study is the thorough phenotypical characterization of participants regarding the quantitative assessment of gut microbiota features and metabolic profile, as well as the controlled randomized design. Together, although the research on gut microbiota at the intersection between diet and human health is mostly based on observational studies, our findings from this 12-wk dietary intervention study support a role for diet in shaping the gut microbiome composition and, for gut microbiota in turn, in modulating diet’s impact on host cardiometabolic markers and its dependence on metabolic and microbial phenotype [57,59,64,65], Furthermore, we found the most pronounced changes on potent SCFA-producing bacteria in this study, which is in line with other studies report convincing associations between SCFAs and diet-induced changes in cardiometabolic health. Despite our novel precision nutrition strategy based on tissue-specific IR phenotypes, this study also has several limitations. First, in contrast to observational studies, which typically use large cohorts, the conclusions from this dietary intervention study are based on a relatively small sample size (*n* = 179). For this reason, the results may need further exploration in future research, which should furthermore include a broader, more diverse multi-ethnic study population. Lastly, although a 12-wk whole-diet intervention may capture diet-induced change in microbiome features, these changes are transient and do not persist permanently [66]. Longer-term studies and other types of metabolic and microbial clustering strategies may be explored to understand and increase the efficacy of new precision nutrition strategies.

In conclusion, a 12-wk HMUFA diet induced more pronounced shifts in overall gut microbiota composition and relative abundances of potent SCFA-producing bacteria specifically in people with LIR. Our study suggests that people with LIR may be more susceptible to diet-induced changes in features of the gut microbiota that are linked to cardiometabolic health improvements. Furthermore, these data confirm that intervention outcomes may be mediated by an individual’s metabolic and related microbial profile. Overall, the findings of this study are in line with the concept of subgroup-based precision nutrition, but the complex interaction between diet, the gut microbiota, and metabolic parameters requires future investigation in studies that include additional cardiometabolic phenotypes, diets with differential macronutrient composition, and that may integrate (multiple) omics data to further investigate underlying mechanisms.

## Author contributions

The authors’ responsibilities were as follows – LAA, GHG, EEB: designed the research; KMJ, IT, AG, GBH, ES: conducted the research; KMJ, AU: analyzed the data and wrote the article; KV: participated in data interpretation and reviewed the manuscript; EEB: had primary responsibility for final content and was the project leader; and all authors: read and approved the final manuscript.

## Data availability

The published article and supplementary information contain the clinical data used to generate the figures in the article. Data generated by 16S rRNA sequencing and corresponding deidentified participant metadata are deposited in the National Center for Biotechnology Information Sequence Read Archive under accession code PRJNA1011518. The complete microbiota analysis pipeline is published at https://github.com/AlexanderUm/PERSON_intervention_microbiome. Any other information required to reanalyze the data reported in this article is available from the lead contact upon reasonable request.

## Funding

Funding for this research was obtained from the industry partners DSM-Firmenich, FrieslandCampina, Danone Nutricia Research, and the Top-sector Agri&Food. EEB obtained funding from Next Food Collective (previously TiFN). The project is organized by and executed under the auspices of Next Food Collective (formerly TiFN), a public-private partnership on precompetitive research in food and nutrition.

## Declaration of generative AI and AI-assisted technologies in the writing process

The authors declare that no generative AI or AI-assisted technologies were used in the writing of the manuscript.

## Conflict of interest

AG is a technical editor for the American Journal of Clinical Nutrition and played no role in the journal’s evaluation of the manuscript. All other authors report no conflicts of interest.
