# Supplementary material for: Gut microbiota responses to isocaloric macronutrient modulation in tissue-specific insulin resistance: a secondary analysis of the PERSonalized glucose Optimization through Nutritional intervention (PERSON) randomized trial
Source: Am J Clin Nutr. 2026 May 5;124(1):101340. doi: 10.1016/j.ajcnut.2026.101340 (PMC13494108; doi:10.1016/j.ajcnut.2026.101340)
Supplement: Supplementary file 1 [file mmc1.docx]

**Gut Microbiota Responses to Isocaloric Macronutrient Modulation in Tissue-Specific Insulin Resistance**

*Jardon et al.*

**Supplementary**

**Material**

**Supplementary Figures**

**Supplementary Figure 1.** CONSORT flow diagram of participant enrollment, allocation and inclusion for analyses. HMUFA, high mono-unsaturated fatty acid diet, LFHP, low-fat, high protein, high fiber diet, LIR, liver insulin resistance; MIR, muscle insulin resistance; OGTT, oral glucose tolerance test.

 **Supplementary Figure 2. Effects of a 12- week HMUFA and LFHP diet on measures of gut microbiota dissimilarity, independent of the individual’s metabolic phenotype.** dbRDA plots using the Unweighted (**A**) and Weighted UniFrac (**B**), Jaccard (**C**), and Bray-Curtis (**D**) indexes, in group following either HMUFA of LFHP diet as indicated in plot title. Model: {distances} ~ Time. The colors and shapes indicate the phenotypes before and after the 12-week intervention. The lines indicate diet-induced intra-individual change. P < 0.05 for statistical significance. HMUFA, high mono-unsaturated fatty acid diet; LFHP, low fat, high protein, high fiber diet; LIR, liver insulin resistance; MIR, muscle insulin resistance; dbRDA, distance -based redundancy analysis.

**Supplementary Figure 3. Effects of 12- week HMUFA and LFHP diet on measures of fecal microbiota diversity and richness.** Microbial diversity and richness as assessed by the Inverse Simpson Index, number of observed taxa, Phylogenetic Diversity and Shannon Index for the full intervention group following the HMUFA or LFHP diet. P < 0.05 for statistical significance. HMUFA, high mono-unsaturated fatty acid diet, LFHP low fat, high protein, high fiber diet.

**Supplementary Figure 4. Effects of 12- week HMUFA and LFHP diet on measures of fecal microbiota diversity and richness in the LIR and MIR phenotypes.** Microbial diversity and richness as assessed by the Inverse Simpson Index, number of observed taxa, Phylogenetic Diversity and Shannon Index for LIR and MIR following the HMUFA or LFHP diet. P < 0.05 for statistical significance. HMUFA, high mono-unsaturated fatty acid diet, LFHP low fat, high protein, high fiber diet; LIR, liver insulin resistance; MIR , muscle insulin resistance.

**Supplementary Figure 5. Effects of a 12-week HMUFA and LFHP diet on individual gut microbial taxa at genus level, using a prevalence of >25%.** The figure depicts genera significantly (q ≤ 0.05) affected by intervention within diet-phenotype (**A**, **B**) or diet (**C**) groups. Explorative genera which may be clinically relevant are shown below (q ≤ 0.1). Figures **A** and **C** show regression coefficients +/- standard error (x-axis) and q-values corresponding to differentially abundant taxa (y-axis) identified by zero-inflated negative binomial regression; color of the dots and error bars indicate if q-value was below (black), or above (grey) set threshold; if the model failed to estimate p-value - no regression coefficient was plotted. Figure **B** shows cumulative median relative abundance of significantly different taxa across a diet-phenotype group; median relative abundance of individual taxa shown by color within a bar as indicated by legend. The taxa prefix corresponds to the last assign taxonomic level of the genus (*G.* – genus, *F.* – family). *q ≤ 0.05. FDR, false discovery rate; HMUFA, high mono-unsaturated fatty acid diet; LFHP, low fat, high protein, high fiber diet; LIR, liver insulin resistance; MIR, muscle insulin resistance.

**A**

**B**

**Supplementary Figure 6. Important Spearman associations between baseline blood metabolism markers and shift in genera abundance from week 0 to week 12 (A) and between the shift in blood glucose metabolism markers and shift in genera abundance from week 0 to week 12 (B).** The scatterplots of ranks visualize correlations considered important (p-values ≤ 0.001 and coefficient ≥ 0.4). Blue line represents a regression fit and results of statistical testing noted in the plot area. CRP, C-reactive protein; HMUFA, high mono-unsaturated fatty acids; HOMA-IR, Homeostatic Model Assessment for Insulin Resistance; LFHP, low-fat high-protein high fiber; LIR, liver insulin resistance; MIR, muscle insulin resistance; MISI, muscle insulin sensitivity index.

**Supplementary Tables**

**Supplementary Table 1. Habitual dietary intake of participants stratified on tissue-specific IR phenotypes within the intervention diets.**

|  | **HMUFA diet** | | **LFHP diet** | | **p-value**  **diet** | **p-value**  **phenotype** |
| --- | --- | --- | --- | --- | --- | --- |
|  | **LIR** | **MIR** | **LIR** | **MIR** |  |  |
| **Habitual dietary intake** | n = 34 | n = 42 | n = 32 | n = 44 |  |  |
| Total energy intake, MJ | 9.7 [7.8, 12.1] | 9.3 [7.8, 10.5] | 9.9 [8.3, 11.9] | 8.2 [7.2, 9.4] | 0.300 | **0.006** |
| Habitual fat intake, en% | 37.7 ± 6.1 | 37.5 ± 6.1 | 36.7 ± 7.3 | 36.4 ± 5.6 | 0.273 | 0.749 |
| Saturated fat, en% | 14.3 ± 3.4 | 13.4 ± 2.3 | 13.6 ± 2.8 | 13.5 ± 2.6 | 0.451 | 0.610 |
| Monounsaturated fat, en% | 13.3 ± 2.2 | 13.4 ± 3.0 | 13.1 ± 3.1 | 12.8 ± 2.4 | 0.330 | 0.836 |
| Polyunsaturated fat, en% | 6.9 ± 1.3 | 7.5 ± 2.0 | 6.8 ± 2.0 | 7.0 ± 1.9 | 0.320 | 0.143 |
| Habitual protein intake, en% | 15.5 ± 2.1 | 15.9 ± 2.5 | 16.0 ± 2.9 | 16.0 ± 2.0 | 0.835 | 0.344 |
| Animal protein, % of total | 59.0 ± 8.1 | 59.4 ± 9.0 | 61.5 ± 10.5 | 60.5 ± 8.7 | 0.269 | 0.711 |
| Plant-based protein, % of total | 41.0 ± 8.0 | 40.6 ± 9.0 | 38.5 ± 10.5 | 39.6 ± 8.7 | 0.265 | 0.710 |
| Habitual CHO intake, en% | 41.4 ± 5.7 | 41.2 ± 6.2 | 41.9 ± 6.8 | 43.0 ± 5.6 | 0.336 | 0.995 |
| Mono- and disaccharides, en% | 17.7 ± 5.5 | 19.3 ± 5.3 | 21.2 ± 7.7 | 20.7 ± 5.1 | **0.028** | 0.608 |
| Polysaccharides, en% | 23.7 ± 5.3 | 21.9 ± 5.3 | 20.6 ± 5.6 | 22.3 ± 4.1 | 0.201 | 0.571 |
| Habitual fiber intake, g/MJ | 2.5 ± 0.5 | 2.6 ± 0.5 | 2.6 ± 0.6 | 2.6 ± 0.7 | 0.930 | 0.897 |
| Habitual alcohol consumption, g | 4.5 [1.1, 13.5] | 7.9 [2.3, 11.2] | 6.3 [1.1, 15.5] | 4.3 [0.8, 9.2] | 0.916 | 0.309 |

Self-reported habitual dietary intake was recorded using a 3-day food frequency questionnaire (FFQ) before the start of the intervention. Differences between the diet groups (p-value diet, HMUFA versus LFHP) and (p-value phenotype, LIR versus MIR at baseline) were assessed using independent samples T-tests for normally distributed numerical data (mean ± SD) and Kruskal-Wallis tests for non-normally distributed numerical data (median [25^th^ percentile, 75^th^ percentile]. P < 0.05 for statistical significance, indicated in bold. En%, energy percentage of total energy intake; HMUFA, high mono-unsaturated fatty acid diet; LFHP, low fat high protein diet; LIR, liver insulin resistance; MIR, muscle insulin resistance; MJ, megajoule; CHO carbohydrates.

**Supplementary Table 2. Effects of the HMUFA and LFHP diets on indicators of cardiometabolic health in LIR and MIR phenotypes Data corresponding to supplementary Figure 3.**

| **HMUFA diet** | | | | | | | | | |
| --- | --- | --- | --- | --- | --- | --- | --- | --- | --- |
|  | **LIR (n = 39)** | | | **MIR (n = 52)** | | | **p-value** | | |
|  | week 0 | week 12 | p-value | week 0 | week 12 | p-value | Phenotype | Time | Phenotype x Time |
| **Glucose metabolism** | | | | | | | | | |
| HOMA-IR, AU | 2.3 ± 0.3 | 2.1 ± 0.3 | **< 0.001** | 1.8 ± 0.1 | 1.8 ± 0.1 | 0.636 | 0.297 | **0.004** | **0.023** |
| Matsuda index, AU | 11.5 ± 1.0 | 14.0 ± 1.2 | **< 0.001** | 13.7 ± 0.7 | 13.8 ± 0.7 | 0.626 | 0.135 | **0.002** | **0.012** |
| MISI, AU | 0.139 ± 0.014 | 0.192 ± 0.022 | **0.007** | 0.136 ± 0.013 | 0.134 ± 0.016 | 0.593 | 0.144 | 0.088 | **0.017** |
| **Other parameters** | | | | | | | | | |
| Body weight (kg) | 93.0 ± 2.6 | 90.7 ± 2.5 | **< 0.001** | 87.2 ± 1.6 | 85.2 ± 1.6 | **< 0.001** | 0.100 | **< 0.001** | 0.862 |
| Body fat (%) | 36.9 ± 1.2 | 35.4 ± 1.3 | **< 0.001** | 37.6 ± 1.2 | 36.3 ± 1.2 | **< 0.001** | 0.634 | **< 0.001** | 0.340 |
| Fasting TG, mmol/L | 1.6 ± 0.1 | 1.4 ± 0.1 | **0.006** | 1.3 ± 0.0 | 1.3 ± 0.0 | 0.895 | 0.100 | **0.031** | **0.046** |
| CRP, mg/L | 1.6 ± 0.2 | 1.5 ± 0.2 | 0.390 | 1.7 ± 0.2 | 1.8 ± 0.2 | 0.727 | 0.249 | 0.676 | 0.381 |
| **LFHP diet** | | | | | | | | | |
|  | **LIR (n = 38)** | | | **MIR (n = 50)** | | | **p-value** | | |
|  | week 0 | week 12 | p-value | week 0 | week 12 | p-value | Phenotype | Time | Phenotype x Time |
| **Glucose metabolism** | | | | | | | | | |
| HOMA-IR, AU | 1.8 ± 0.2 | 1.8 ± 0.2 | 0.163 | 1.8 ± 0.2 | 1.5 ± 0.1 | **0.001** | 0.619 | **0.002** | 0.262 |
| Matsuda index, AU | 13.6 ± 1.1 | 14.9 ± 1.2 | 0.125 | 13.8 ± 1.1 | 17.0 ± 1.2 | **< 0.001** | 0.701 | **< 0.001** | 0.077 |
| MISI, AU | 0.179 ± 0.018 | 0.180 ± 0.016 | 0.630 | 0.144 ± 0.011 | 0.185 ± 0.020 | 0.055 | 0.249 | **0.102** | 0.355 |
| **Other parameters** |  |  |  |  |  |  |  |  |  |
| Body weight (kg) | 91.3 ± 2.4 | 89.3 ± 2.4 | **< 0.001** | 84.7 ± 1.3 | 82.4 ± 1.3 | **< 0.001** | 0.147 | **< 0.001** | 0.275 |
| Body fat (%) | 35.2 ± 1.0 | 34.2 ± 1.2 | **< 0.001** | 38.8 ± 0.9 | 37.1 ± 0.9 | **< 0.001** | **0.030** | **< 0.001** | 0.233 |
| Fasting TG, mmol/L | 1.5 ± 0.1 | 1.3 ± 0.0 | **0.024** | 1.6 ± 0.1 | 1.4 ± 0.0 | **< 0.001** | 0.926 | **< 0.001** | 0.484 |
| CRP, mg/L | 1.2 ± 0.2 | 1.1 ± 0.2 | 0.411 | 1.9 ± 0.2 | 1.4 ± 0.2 | **< 0.001** | 0.027 | **0.002** | **0.046** |

Data are as mean ± standard error of mean (SEM). Data were analyzed using a mixed model with repeated measures, stratified per diet. Models were adjusted for age, sex, and study center. Significant p-values (< 0.05) are highlighted in bold. BMI, body mass index; CRP, C-reactive protein; HMUFA, high mono-unsaturated fatty acids; HOMA-IR, Homeostatic Model Assessment for Insulin Resistance; LFHP, low-fat high-protein high fiber; LIR, liver insulin resistance; MIR, muscle insulin resistance; MISI, muscle insulin sensitivity index; TG, triglyceride.

**Supplementary Table 3. ADONIS test results of gut microbiota community change after 12-week HMUFA and LFHP diets in participants with predominant liver or muscle insulin resistance stratified by diet type. Data corresponding to Supplementary Figure 2.**

| **Term** | **Degrees of Freedom** | **Sum of Squares** | **R2** | **F-statistics** | **P-value** | **Dispersion - F** | **Dispersion - P** |
| --- | --- | --- | --- | --- | --- | --- | --- |
| **HMUFA: Unweighted UniFrac** | | | | | | | |
| **TimeNumeric** | 1 | 0.092 | 0.003 | 0.653 | 0.017 | 0.312 | 0.433 |
| **Residual** | 196 | 27.652 | 0.997 |  |  |  |  |
| **Total** | 197 | 27.744 | 1 |  |  |  |  |
| **HMUFA: Weighted UniFrac** | | | | | | | |
| **TimeNumeric** | 1 | 0.017 | 0.004 | 0.784 | 0.004 | < 0.001 | 1 |
| **Residual** | 196 | 4.316 | 0.996 |  |  |  |  |
| **Total** | 197 | 4.333 | 1 |  |  |  |  |
| **HMUFA: Jaccard** | | | | | | | |
| **TimeNumeric** | 1 | 0.176 | 0.003 | 0.558 | 0.001 | 0.076 | 0.671 |
| **Residual** | 196 | 61.884 | 0.997 |  |  |  |  |
| **Total** | 197 | 62.06 | 1 |  |  |  |  |
| **HMUFA: Bray-Curtis** | | | | | | | |
| **TimeNumeric** | 1 | 0.129 | 0.003 | 0.573 | < 0.001 | 0.031 | 0.796 |
| **Residual** | 196 | 44.042 | 0.997 |  |  |  |  |
| **Total** | 197 | 44.17 | 1 |  |  |  |  |
| **LFHP: Unweighted UniFrac** | | | | | | | |
| **TimeNumeric** | 1 | 0.059 | 0.002 | 0.39 | 0.502 | 0.541 | 0.19 |
| **Residual** | 180 | 26.959 | 0.998 |  |  |  |  |
| **Total** | 181 | 27.018 | 1 |  |  |  |  |
| **LFHP: Weighted UniFrac** | | | | | | | |
| **TimeNumeric** | 1 | 0.01 | 0.002 | 0.423 | 0.109 | 0.101 | 0.556 |
| **Residual** | 180 | 4.171 | 0.998 |  |  |  |  |
| **Total** | 181 | 4.181 | 1 |  |  |  |  |
| **LFHP: Jaccard** | | | | | | | |
| **TimeNumeric** | 1 | 0.136 | 0.002 | 0.42 | 0.461 | 0.499 | 0.149 |
| **Residual** | 180 | 58.5 | 0.998 |  |  |  |  |
| **Total** | 181 | 58.636 | 1 |  |  |  |  |
| **LFHP: Bray-Curtis** | | | | | | | |
| **TimeNumeric** | 1 | 0.077 | 0.002 | 0.325 | 0.194 | 0.014 | 0.819 |
| **Residual** | 180 | 42.479 | 0.998 |  |  |  |  |
| **Total** | 181 | 42.556 | 1 |  |  |  |  |

Unweighted and Weighted UniFrac, Jaccard, and Bray-Curtis distances between week 0 and week 12 of flowing a HMUFA or LFHP diet. *ADONIS* model: distances ~ ParticipantID + Time with 9999 permutation blocked by participant ID. Statistical significance at p < 0.05. HMUFA, high mono-unsaturated fatty acid diet; LIR, liver insulin resistance; MIR, muscle insulin resistance.

**Supplementary Table 4. ADONIS test results of gut microbiota community change after 12-week HMUFA and LFHP diets in participants with predominant liver or muscle insulin resistance stratified by diet type and phenotype. Data corresponding to Figure 1.**

| **Term** | **Degrees of Freedom** | **Sum of Squares** | **R2** | **F-stats** | **P-value** | **Dispersion - F** | **Dispersion - P** |
| --- | --- | --- | --- | --- | --- | --- | --- |
| **HMUFA-LIR: Unweighted UniFrac** | | | | | | | |
| **TimeNumeric** | 1 | 0.104 | 0.009 | 0.752 | 0.002 | 0.965 | 0.063 |
| **Residual** | 80 | 11.087 | 0.991 |  |  |  |  |
| **Total** | 81 | 11.191 | 1 |  |  |  |  |
| **HMUFA-LIR: Weighted UniFrac** | | | | | | | |
| **TimeNumeric** | 1 | 0.021 | 0.012 | 0.985 | < 0.001 | 0.681 | 0.215 |
| **Residual** | 80 | 1.67 | 0.988 |  |  |  |  |
| **Total** | 81 | 1.69 | 1 |  |  |  |  |
| **HMUFA-LIR: Jaccard** | | | | | | | |
| **TimeNumeric** | 1 | 0.158 | 0.006 | 0.506 | 0.006 | 0.167 | 0.492 |
| **Residual** | 80 | 24.922 | 0.994 |  |  |  |  |
| **Total** | 81 | 25.08 | 1 |  |  |  |  |
| **HMUFA-LIR: Bray-Curtis** | | | | | | | |
| **TimeNumeric** | 1 | 0.108 | 0.006 | 0.497 | < 0.001 | 0.002 | 0.945 |
| **Residual** | 80 | 17.469 | 0.994 |  |  |  |  |
| **Total** | 81 | 17.578 | 1 |  |  |  |  |
| **HMUFA-MIR: Unweighted UniFrac** | | | | | | | |
| **TimeNumeric** | 1 | 0.051 | 0.003 | 0.36 | 0.672 | 0.022 | 0.863 |
| **Residual** | 114 | 16.261 | 0.997 |  |  |  |  |
| **Total** | 115 | 16.312 | 1 |  |  |  |  |
| **HMUFA-MIR: Weighted UniFrac** | | | | | | | |
| **TimeNumeric** | 1 | 0.007 | 0.003 | 0.325 | 0.615 | 0.279 | 0.621 |
| **Residual** | 114 | 2.592 | 0.997 |  |  |  |  |
| **Total** | 115 | 2.6 | 1 |  |  |  |  |
| **HMUFA-MIR: Jaccard** | | | | | | | |
| **TimeNumeric** | 1 | 0.135 | 0.004 | 0.424 | 0.219 | 0.002 | 0.952 |
| **Residual** | 114 | 36.305 | 0.996 |  |  |  |  |
| **Total** | 115 | 36.44 | 1 |  |  |  |  |
| **HMUFA-MIR: Bray-Curtis** | | | | | | | |
| **TimeNumeric** | 1 | 0.076 | 0.003 | 0.33 | 0.129 | 0.056 | 0.748 |
| **Residual** | 114 | 26.102 | 0.997 |  |  |  |  |
| **Total** | 115 | 26.178 | 1 |  |  |  |  |
| **LFHP-LIR: Unweighted UniFrac** | | | | | | | |
| **TimeNumeric** | 1 | 0.038 | 0.004 | 0.268 | 0.924 | 0.089 | 0.595 |
| **Residual** | 76 | 10.731 | 0.997 |  |  |  |  |
| **Total** | 77 | 10.769 | 1 |  |  |  |  |
| **LFHP-LIR: Weighted UniFrac** | | | | | | | |
| **TimeNumeric** | 1 | 0.005 | 0.003 | 0.224 | 0.735 | 0.43 | 0.225 |
| **Residual** | 76 | 1.727 | 0.997 |  |  |  |  |
| **Total** | 77 | 1.732 | 1 |  |  |  |  |
| **LFHP-LIR: Jaccard** | | | | | | | |
| **TimeNumeric** | 1 | 0.127 | 0.005 | 0.401 | 0.232 | 0.021 | 0.775 |
| **Residual** | 76 | 24.017 | 0.995 |  |  |  |  |
| **Total** | 77 | 24.144 | 1 |  |  |  |  |
| **LFHP-LIR: Bray-Curtis** | | | | | | | |
| **TimeNumeric** | 1 | 0.07 | 0.004 | 0.31 | 0.105 | 0.838 | 0.095 |
| **Residual** | 76 | 17.195 | 0.996 |  |  |  |  |
| **Total** | 77 | 17.265 | 1 |  |  |  |  |
| **LFHP-MIR: Unweighted UniFrac** | | | | | | | |
| **TimeNumeric** | 1 | 0.066 | 0.004 | 0.425 | 0.418 | 0.478 | 0.254 |
| **Residual** | 102 | 15.953 | 0.996 |  |  |  |  |
| **Total** | 103 | 16.02 | 1 |  |  |  |  |
| **LFHP-MIR: Weighted UniFrac** | | | | | | | |
| **TimeNumeric** | 1 | 0.009 | 0.004 | 0.392 | 0.156 | 0.672 | 0.114 |
| **Residual** | 102 | 2.404 | 0.996 |  |  |  |  |
| **Total** | 103 | 2.413 | 1 |  |  |  |  |
| **LFHP-MIR: Jaccard** | | | | | | | |
| **TimeNumeric** | 1 | 0.147 | 0.004 | 0.445 | 0.492 | 0.863 | 0.068 |
| **Residual** | 102 | 33.752 | 0.996 |  |  |  |  |
| **Total** | 103 | 33.899 | 1 |  |  |  |  |
| **LFHP-MIR: Bray-Curtis** | | | | | | | |
| **TimeNumeric** | 1 | 0.075 | 0.003 | 0.31 | 0.443 | 0.395 | 0.198 |
| **Residual** | 102 | 24.749 | 0.997 |  |  |  |  |
| **Total** | 103 | 24.824 | 1 |  |  |  |  |

Unweighted and Weighted UniFrac, Jaccard, and Bray-Curtis distances in LIR and MIR phenotypes following HMUFA or LFHP diet. *ADONIS* model: distances ~ ParticipantID + Time with 9999 permutation blocked by participant ID. Statistical significance at p < 0.05. HMUFA, high mono-unsaturated fatty acid diet; LIR, liver insulin resistance; MIR, muscle insulin resistance.

**Supplementary Table 5. Summary of alpha diversity indices and Wilcoxon paired test results for shifts stratified by diet type.**

|  | **Mean ±SD(%)** | **Mean ±SD(%)** | **Median[IQR:25%-75%](%)** | **Median[IQR:25%-75%](%)** | **Statsistics (W)** | **P-value** |
| --- | --- | --- | --- | --- | --- | --- |
| **Diet** | **Week 0** | **Week 12** | **Week 0** | **Week 12** |  |  |
| **Observed Taxa** | | | | | | |
| **HMUFA** | 256.162 ±72.246 | 261.515 ±66.147 | 260.000[205.000-300.500] | 262.000[219.000-302.500] | 2237.5 | 0.506 |
| **LFHP** | 264.275 ±84.456 | 255.462 ±75.012 | 257.000[201.000-317.000] | 248.000[195.000-298.500] | 2301.5 | 0.41 |
| **Shannon Index** | | | | | | |
| **HMUFA** | 4.412 ±0.356 | 4.386 ±0.383 | 4.472[4.223-4.637] | 4.438[4.214-4.645] | 2671 | 0.495 |
| **LFHP** | 4.426 ±0.382 | 4.367 ±0.391 | 4.496[4.241-4.699] | 4.409[4.094-4.636] | 2509 | 0.1 |
| **Inverse Simpson** | | | | | | |
| **HMUFA** | 44.737 ±18.157 | 42.594 ±16.435 | 43.918[30.498-53.738] | 42.496[31.415-52.369] | 2913 | 0.127 |
| **LFHP** | 45.047 ±18.694 | 42.233 ±17.914 | 42.155[30.898-56.440] | 40.104[28.710-53.323] | 2433 | 0.179 |
| **Phylogenetic Diversity** | | | | | | |
| **HMUFA** | 23.711 ±5.326 | 23.839 ±5.284 | 24.122[19.754-27.647] | 24.123[19.765-27.231] | 2325 | 0.602 |
| **LFHP** | 23.717 ±6.071 | 23.136 ±5.628 | 24.057[19.144-27.732] | 23.341[18.897-27.330] | 2364 | 0.284 |

ASV count per sample was normalized by rarefaction prior to calculation. Diet effects on alpha diversity were assessed by Wilcoxon rank sum test with continuity correction. Statistical significance at p < 0.05. LFHP, low fat high protein, high fiber diet.

**Supplementary Table 6. Summary of alpha diversity indices for shifts stratified by diet type and individual diet x phenotype strata. Data corresponding to Figure 2.**

| **Term** | **Estimates** | **Standard Errors** | **p-values** | **Index** |
| --- | --- | --- | --- | --- |
| **HMUFA** | | | | |
| **TimeNumeric** | 0.000 | 0.001 | 0.949 | Shannon |
| **TimeNumeric** | 0.000 | 0.000 | 0.623 | Simpson |
| **LFHP** | | | | |
| **TimeNumeric** | -0.001 | 0.001 | 0.643 | Shannon |
| **TimeNumeric** | -0.001 | 0.000 | 0.065 | Simpson |
| **HMUFA-LIR** | | | | |
| **TimeNumeric** | -0.001 | 0.002 | 0.617 | Shannon |
| **TimeNumeric** | -0.001 | 0.001 | 0.111 | Simpson |
| **HMUFA-MIR** | | | | |
| **TimeNumeric** | 0.002 | 0.002 | 0.246 | Shannon |
| **TimeNumeric** | 0.000 | 0.000 | 0.336 | Simpson |
| **LFHP-LIR** | | | | |
| **TimeNumeric** | -0.007 | 0.002 | 0.000 | Shannon |
| **TimeNumeric** | 0.000 | 0.001 | 0.995 | Simpson |
| **LFHP-MIR** | | | | |
| **TimeNumeric** | 0.003 | 0.002 | 0.158 | Shannon |
| **TimeNumeric** | -0.001 | 0.001 | 0.060 | Simpson |

Shannon and Simpson indexes were calculated at genus level without prior count normalization as implemented in the DivNet method, followed by statistical testing will linear mixed models as implemented in the breakaway package to assess effect of time with participant ID as random effect. P < 0.05 was considered statistically significant. HMUFA, high mono-unsaturated fatty acid diet; LFHP, low-fat high-protein high fiber diet; LIR, liver insulin resistance; MIR, muscle insulin resistance.

**Supplementary Table 7. Changes in individual fecal microbial genus abundances after a 12-week HMUFA or LFHP diet, related to Figure 3. Prevalence cutoff >50%.**

| **Taxa** | **Mean ±SD(%)** | **Mean ±SD(%)** | **Median[IQR:25%-75%](%)** | **Median[IQR:25%-75%](%)** | **Coef** | **SE** | **P-value** | **Q-value** |
| --- | --- | --- | --- | --- | --- | --- | --- | --- |
|  | **{Week 0}** | **{Week 12}** | **{Week 0}** | **{Week 12}** |  |  |  |  |
| **HMUFA** | | | | | | | | |
| ***G. Turicibacter*** | 0.115 ±0.227 | 0.111 ±0.216 | 0.009[0.000-0.079] | 0.025[0.000-0.089] | -0.005 | 0.017 | 0.772 | 0.863 |
| ***G. Akkermansia*** | 1.968 ±3.094 | 1.962 ±2.848 | 0.498[0.099-2.686] | 0.815[0.112-2.760] | 0.011 | 0.016 | 0.489 | 0.685 |
| ***G. Muribaculaceae*** | 0.818 ±2.395 | 1.040 ±2.577 | 0.000[0.000-0.476] | 0.006[0.000-0.857] | 0.055 | 0.015 | < 0.001 | 0.004 |
| ***G. Dialister*** | 1.012 ±2.266 | 0.990 ±2.152 | 0.025[0.000-1.087] | 0.016[0.000-1.168] | 0.011 | 0.011 | 0.356 | 0.607 |
| ***F. Oscillospiraceae-003*** | 0.336 ±0.403 | 0.476 ±0.528 | 0.207[0.073-0.486] | 0.338[0.122-0.692] | 0.036 | 0.009 | < 0.001 | 0.002 |
| ***G. Prevotella*** | 3.634 ±6.940 | 4.338 ±7.922 | 0.270[0.049-3.651] | 0.584[0.076-4.823] | 0.036 | 0.015 | 0.016 | 0.084 |
| ***G. Sutterella*** | 0.377 ±0.713 | 0.433 ±0.871 | 0.162[0.042-0.325] | 0.183[0.063-0.461] | 0.032 | 0.013 | 0.012 | 0.072 |
| ***G. Eubacterium eligens group*** | 0.298 ±0.673 | 0.351 ±0.538 | 0.124[0.040-0.280] | 0.156[0.049-0.448] | 0.028 | 0.01 | 0.005 | 0.052 |
| ***G. Lachnospiraceae UCG-010*** | 0.099 ±0.149 | 0.143 ±0.234 | 0.032[0.008-0.139] | 0.050[0.010-0.194] | 0.027 | 0.011 | 0.011 | 0.072 |
| ***G. Lachnospiraceae NK4A136 group*** | 1.615 ±1.828 | 2.063 ±1.954 | 1.091[0.546-2.157] | 1.444[0.706-2.825] | 0.027 | 0.008 | 0.001 | 0.012 |
| ***G. Eubacterium xylanophilum group*** | 0.216 ±0.231 | 0.289 ±0.310 | 0.152[0.034-0.329] | 0.220[0.065-0.387] | 0.025 | 0.01 | 0.01 | 0.069 |
| ***G. Lachnospira*** | 0.183 ±0.236 | 0.261 ±0.475 | 0.111[0.047-0.238] | 0.135[0.062-0.313] | 0.024 | 0.009 | 0.01 | 0.069 |
| ***G. Coprococcus*** | 1.957 ±1.648 | 1.602 ±1.412 | 1.403[0.811-2.614] | 1.064[0.521-2.237] | -0.017 | 0.007 | 0.02 | 0.091 |
| ***G. Blautia*** | 5.106 ±4.140 | 4.159 ±3.953 | 3.690[2.277-6.525] | 2.913[1.483-5.569] | -0.018 | 0.007 | 0.008 | 0.069 |
| ***G. Dorea*** | 1.417 ±1.132 | 1.140 ±1.204 | 1.174[0.677-1.683] | 0.757[0.382-1.544] | -0.022 | 0.008 | 0.007 | 0.06 |
| ***G. Enterorhabdus*** | 0.082 ±0.177 | 0.054 ±0.091 | 0.026[0.000-0.083] | 0.015[0.000-0.069] | -0.027 | 0.011 | 0.017 | 0.085 |
| ***G. Candidatus Soleaferrea*** | 0.009 ±0.019 | 0.005 ±0.012 | 0.002[0.000-0.010] | 0.000[0.000-0.006] | -0.028 | 0.012 | 0.016 | 0.084 |
| ***F. Lachnospiraceae-900066575*** | 0.023 ±0.030 | 0.021 ±0.026 | 0.014[0.000-0.037] | 0.017[0.000-0.032] | -0.004 | 0.009 | 0.654 | 0.801 |
| ***G. Erysipelotrichaceae UCG-003*** | 0.795 ±1.053 | 0.535 ±0.655 | 0.444[0.135-0.930] | 0.303[0.071-0.675] | -0.033 | 0.009 | < 0.001 | 0.004 |
| ***G. Senegalimassilia*** | 0.117 ±0.446 | 0.090 ±0.352 | 0.016[0.000-0.094] | 0.000[0.000-0.049] | -0.035 | 0.011 | 0.002 | 0.028 |
| ***G. Escherichia-Shigella*** | 0.641 ±1.888 | 0.692 ±3.126 | 0.050[0.012-0.134] | 0.042[0.009-0.102] | -0.035 | 0.015 | 0.016 | 0.084 |
| ***G. Bifidobacterium*** | 1.110 ±1.464 | 0.877 ±1.312 | 0.601[0.178-1.262] | 0.319[0.077-1.231] | -0.022 | 0.012 | 0.058 | 0.181 |
| ***F. Eggerthellaceae*** | 0.271 ±0.503 | 0.166 ±0.316 | 0.060[0.000-0.340] | 0.033[0.000-0.184] | -0.037 | 0.012 | 0.001 | 0.021 |
| ***G. Holdemanella*** | 1.119 ±2.237 | 0.703 ±1.417 | 0.031[0.000-0.925] | 0.022[0.000-0.758] | -0.03 | 0.016 | 0.069 | 0.189 |
| ***G. Ruminococcus torques group*** | 0.739 ±1.062 | 0.429 ±0.528 | 0.453[0.192-0.954] | 0.265[0.117-0.531] | -0.042 | 0.008 | < 0.001 | < 0.001 |
| ***G. Adlercreutzia*** | 0.127 ±0.268 | 0.088 ±0.170 | 0.000[0.000-0.125] | 0.007[0.000-0.083] | -0.044 | 0.015 | 0.003 | 0.035 |
| ***G. Collinsella*** | 0.448 ±0.556 | 0.286 ±0.433 | 0.271[0.061-0.662] | 0.139[0.057-0.315] | -0.045 | 0.008 | < 0.001 | < 0.001 |
| **LFHP** | | | | | | | | |
| ***G. Turicibacter*** | 0.097 ±0.192 | 0.257 ±0.498 | 0.002[0.000-0.071] | 0.015[0.000-0.191] | 0.094 | 0.023 | < 0.001 | 0.002 |
| ***G. Akkermansia*** | 1.841 ±3.251 | 2.430 ±4.314 | 0.236[0.041-3.021] | 0.573[0.048-3.036] | 0.062 | 0.013 | < 0.001 | < 0.001 |
| ***G. Muribaculaceae*** | 0.788 ±1.781 | 0.964 ±1.920 | 0.004[0.000-0.531] | 0.025[0.000-1.084] | 0.029 | 0.022 | 0.179 | 0.455 |
| ***G. Dialister*** | 1.030 ±1.838 | 1.455 ±2.485 | 0.032[0.000-1.170] | 0.068[0.000-1.990] | 0.043 | 0.015 | 0.004 | 0.063 |
| ***F. Oscillospiraceae-003*** | 0.312 ±0.451 | 0.400 ±0.601 | 0.104[0.038-0.388] | 0.187[0.057-0.542] | 0.029 | 0.013 | 0.023 | 0.214 |
| ***G. Prevotella*** | 5.291 ±9.054 | 5.233 ±8.453 | 0.816[0.034-6.877] | 1.138[0.155-7.053] | 0.011 | 0.015 | 0.454 | 0.792 |
| ***G. Sutterella*** | 0.292 ±0.402 | 0.241 ±0.344 | 0.122[0.038-0.362] | 0.098[0.018-0.338] | -0.011 | 0.014 | 0.417 | 0.778 |
| ***G. Eubacterium eligens group*** | 0.227 ±0.352 | 0.252 ±0.345 | 0.081[0.024-0.274] | 0.134[0.051-0.299] | 0.053 | NA | NA | NA |
| ***G. Lachnospiraceae UCG-010*** | 0.120 ±0.318 | 0.103 ±0.170 | 0.037[0.000-0.125] | 0.028[0.000-0.135] | 0.012 | 0.014 | 0.414 | 0.778 |
| ***G. Lachnospiraceae NK4A136 group*** | 1.363 ±1.124 | 1.476 ±1.753 | 1.102[0.472-1.893] | 1.012[0.506-1.850] | 0.005 | 0.008 | 0.521 | 0.811 |
| ***G. Eubacterium xylanophilum group*** | 0.223 ±0.293 | 0.225 ±0.312 | 0.110[0.029-0.335] | 0.109[0.034-0.268] | 0.002 | 0.012 | 0.886 | 0.947 |
| ***G. Lachnospira*** | 0.218 ±0.317 | 0.194 ±0.237 | 0.105[0.045-0.253] | 0.109[0.032-0.257] | 0.006 | 0.013 | 0.62 | 0.866 |
| ***G. Coprococcus*** | 1.902 ±1.593 | 1.917 ±1.517 | 1.323[0.739-2.733] | 1.556[0.639-3.093] | 0.001 | 0.007 | 0.838 | 0.94 |
| ***G. Blautia*** | 5.620 ±4.900 | 6.104 ±6.385 | 4.119[2.250-7.341] | 4.013[1.974-7.782] | 0.004 | 0.007 | 0.566 | 0.851 |
| ***G. Dorea*** | 1.343 ±1.091 | 1.293 ±1.038 | 1.053[0.538-1.661] | 1.070[0.561-1.722] | 0.001 | 0.007 | 0.864 | 0.94 |
| ***G. Enterorhabdus*** | 0.073 ±0.138 | 0.061 ±0.125 | 0.015[0.000-0.075] | 0.007[0.000-0.084] | -0.02 | 0.016 | 0.193 | 0.478 |
| ***G. Candidatus Soleaferrea*** | 0.007 ±0.013 | 0.005 ±0.008 | 0.003[0.000-0.009] | 0.001[0.000-0.006] | 0.05 | NA | NA | NA |
| ***F. Lachnospiraceae-900066575*** | 0.022 ±0.027 | 0.016 ±0.020 | 0.015[0.000-0.038] | 0.011[0.000-0.022] | -0.032 | 0.01 | 0.001 | 0.017 |
| ***G. Erysipelotrichaceae UCG-003*** | 0.665 ±1.077 | 0.717 ±1.145 | 0.239[0.104-0.747] | 0.335[0.141-0.688] | 0.019 | 0.01 | 0.06 | 0.327 |
| ***G. Senegalimassilia*** | 0.069 ±0.131 | 0.047 ±0.076 | 0.018[0.000-0.102] | 0.005[0.000-0.060] | -0.023 | 0.011 | 0.026 | 0.218 |
| ***G. Escherichia-Shigella*** | 0.845 ±3.251 | 0.932 ±3.277 | 0.030[0.000-0.173] | 0.031[0.005-0.163] | 0.022 | NA | NA | NA |
| ***G. Bifidobacterium*** | 1.071 ±1.175 | 0.717 ±0.866 | 0.725[0.260-1.433] | 0.401[0.110-1.050] | -0.037 | 0.011 | 0.001 | 0.017 |
| ***F. Eggerthellaceae*** | 0.260 ±0.494 | 0.235 ±0.657 | 0.043[0.000-0.334] | 0.058[0.000-0.228] | -0.021 | 0.013 | 0.102 | 0.367 |
| ***G. Holdemanella*** | 1.194 ±1.913 | 0.931 ±1.939 | 0.234[0.000-1.658] | 0.102[0.000-0.928] | -0.039 | 0.013 | 0.002 | 0.035 |
| ***G. Ruminococcus torques group*** | 0.676 ±0.743 | 0.571 ±0.662 | 0.487[0.170-0.829] | 0.373[0.171-0.752] | -0.006 | 0.01 | 0.498 | 0.796 |
| ***G. Adlercreutzia*** | 0.095 ±0.215 | 0.083 ±0.196 | 0.000[0.000-0.068] | 0.000[0.000-0.052] | -0.015 | 0.013 | 0.242 | 0.542 |
| ***G. Collinsella*** | 0.477 ±0.730 | 0.392 ±0.489 | 0.283[0.103-0.529] | 0.237[0.066-0.519] | -0.013 | 0.01 | 0.176 | 0.455 |

Intervention effects are indicated by the model coefficient and standard error of MaAsLin2 statistical models. Model coefficients represent the effect size, positive coefficients indicate an increased relative abundance of a specific genus. Only significant, diet-induced changes with FDR adjusted p-value (q-values) equal or less than 0.05 considered statistically different. ASV, amplicon sequence variant; FDR, false discovery rate; HMUFA, high mono-unsaturated fatty acid diet;LFHP, low fat, high protein, high fiber diet.

**Supplementary Table 8. Changes in individual fecal microbial genus abundances after a 12-week HMUFA or LFHP diet that were significant in tissue-specific LIR or MIR phenotypes, related to Figure 3. Prevalence cutoff >50%.**

| **Taxa** | **Mean ±SD(%)** | **Mean ±SD(%)** | **Median[IQR:25%-75%](%)** | **Median[IQR:25%-75%](%)** | **Coef** | **SE** | **P-value** | **Q-value** |
| --- | --- | --- | --- | --- | --- | --- | --- | --- |
|  | **{Week 0}** | **{Week 12}** | **{Week 0}** | **{Week 12}** |  |  |  |  |
| **HMUFA-LIR** | | | | | | | | |
| ***G. Terrisporobacter*** | 0.212 ±0.527 | 0.215 ±0.415 | 0.000[0.000-0.122] | 0.020[0.000-0.211] | 0.031 | 0.017 | 0.074 | 0.223 |
| ***G. Turicibacter*** | 0.100 ±0.175 | 0.105 ±0.165 | 0.008[0.000-0.119] | 0.032[0.004-0.091] | 0.001 | 0.026 | 0.956 | 0.984 |
| ***G. Akkermansia*** | 1.234 ±2.032 | 1.664 ±2.130 | 0.332[0.095-1.418] | 0.835[0.128-2.538] | 0.04 | 0.023 | 0.082 | 0.234 |
| ***G. Muribaculaceae*** | 0.938 ±2.863 | 0.886 ±2.777 | 0.000[0.000-0.301] | 0.006[0.000-0.761] | 0.036 | 0.024 | 0.133 | 0.299 |
| ***G. Butyricimonas*** | 0.157 ±0.165 | 0.255 ±0.282 | 0.108[0.033-0.232] | 0.152[0.043-0.353] | 0.045 | 0.016 | 0.004 | 0.046 |
| ***F. Oscillospiraceae-003*** | 0.337 ±0.449 | 0.433 ±0.355 | 0.180[0.081-0.514] | 0.346[0.126-0.627] | 0.033 | 0.015 | 0.024 | 0.131 |
| ***G. Odoribacter*** | 0.329 ±0.344 | 0.515 ±0.760 | 0.179[0.102-0.404] | 0.271[0.121-0.543] | 0.036 | 0.013 | 0.006 | 0.054 |
| ***G. Alistipes*** | 4.950 ±3.852 | 6.361 ±4.413 | 4.399[2.097-6.989] | 5.268[3.210-7.590] | 0.03 | 0.008 | < 0.001 | 0.006 |
| ***G. Lachnospiraceae NK4A136 group*** | 2.090 ±2.500 | 2.540 ±2.303 | 1.274[0.762-2.265] | 2.500[0.767-3.804] | 0.03 | 0.014 | 0.03 | 0.152 |
| ***F. Oscillospiraceae-002*** | 2.425 ±1.970 | 3.072 ±2.441 | 2.210[0.834-3.146] | 2.728[1.397-4.100] | 0.026 | 0.009 | 0.004 | 0.046 |
| ***F. Oscillospiraceae-005*** | 0.732 ±0.891 | 0.861 ±1.149 | 0.435[0.246-0.906] | 0.601[0.251-1.071] | 0.02 | 0.008 | 0.015 | 0.095 |
| ***G. Escherichia-Shigella*** | 0.661 ±2.040 | 0.399 ±1.483 | 0.047[0.008-0.138] | 0.046[0.007-0.078] | -0.032 | NA | NA | NA |
| ***G. Blautia*** | 5.912 ±4.891 | 4.270 ±4.601 | 4.011[2.734-6.762] | 2.684[1.379-5.788] | -0.029 | 0.009 | 0.001 | 0.014 |
| ***G. Dorea*** | 1.528 ±1.241 | 1.005 ±0.895 | 1.192[0.645-1.731] | 0.693[0.335-1.357] | -0.032 | 0.01 | 0.002 | 0.027 |
| ***G. Enterorhabdus*** | 0.092 ±0.215 | 0.050 ±0.103 | 0.038[0.000-0.085] | 0.015[0.000-0.067] | -0.038 | 0.014 | 0.005 | 0.052 |
| ***G. Erysipelatoclostridium*** | 0.039 ±0.054 | 0.053 ±0.136 | 0.004[0.000-0.082] | 0.022[0.000-0.051] | -0.018 | 0.015 | 0.248 | 0.429 |
| ***G. Erysipelotrichaceae UCG-003*** | 0.842 ±1.103 | 0.511 ±0.640 | 0.458[0.216-0.941] | 0.311[0.080-0.640] | -0.041 | 0.012 | 0.001 | 0.014 |
| ***F. Christensenellaceae*** | 0.016 ±0.023 | 0.019 ±0.024 | 0.007[0.000-0.022] | 0.010[0.000-0.026] | 0.023 | 0.018 | 0.203 | 0.394 |
| ***G. Bifidobacterium*** | 1.421 ±1.652 | 1.033 ±1.349 | 0.725[0.373-1.953] | 0.337[0.139-1.529] | -0.016 | 0.017 | 0.357 | 0.513 |
| ***G. Ruminococcus gauvreauii group*** | 0.313 ±0.387 | 0.157 ±0.159 | 0.195[0.089-0.353] | 0.128[0.031-0.204] | -0.043 | 0.013 | 0.001 | 0.014 |
| ***F. Eggerthellaceae*** | 0.260 ±0.638 | 0.118 ±0.276 | 0.014[0.000-0.197] | 0.014[0.000-0.106] | -0.05 | 0.02 | 0.012 | 0.083 |
| ***G. Ruminococcus torques group*** | 0.604 ±0.485 | 0.336 ±0.352 | 0.458[0.248-0.965] | 0.203[0.115-0.398] | -0.05 | 0.012 | < 0.001 | 0.001 |
| ***G. Adlercreutzia*** | 0.130 ±0.307 | 0.090 ±0.186 | 0.000[0.000-0.102] | 0.000[0.000-0.039] | -0.056 | 0.021 | 0.008 | 0.069 |
| ***G. Romboutsia*** | 0.581 ±0.622 | 0.391 ±0.483 | 0.350[0.116-0.898] | 0.167[0.042-0.654] | -0.04 | 0.015 | 0.01 | 0.073 |
| ***G. Eubacterium ventriosum group*** | 0.249 ±0.256 | 0.277 ±0.272 | 0.159[0.070-0.319] | 0.193[0.088-0.388] | 0.012 | 0.013 | 0.368 | 0.513 |
| ***G. Collinsella*** | 0.495 ±0.498 | 0.276 ±0.378 | 0.370[0.161-0.657] | 0.155[0.082-0.307] | -0.062 | 0.012 | < 0.001 | < 0.001 |
| **HMUFA-MIR** | | | | | | | | |
| ***G. Terrisporobacter*** | 0.170 ±0.561 | 0.321 ±1.112 | 0.000[0.000-0.066] | 0.000[0.000-0.099] | 0.064 | NA | NA | NA |
| ***G. Turicibacter*** | 0.125 ±0.259 | 0.115 ±0.247 | 0.012[0.000-0.068] | 0.018[0.000-0.085] | 0.002 | 0.024 | 0.949 | 0.966 |
| ***G. Akkermansia*** | 2.487 ±3.591 | 2.173 ±3.264 | 0.737[0.111-4.038] | 0.745[0.110-3.273] | -0.006 | 0.021 | 0.783 | 0.961 |
| ***G. Muribaculaceae*** | 0.734 ±2.024 | 1.149 ±2.445 | 0.004[0.000-0.656] | 0.004[0.000-0.991] | 0.063 | 0.017 | < 0.001 | 0.014 |
| ***G. Butyricimonas*** | 0.199 ±0.216 | 0.167 ±0.202 | 0.133[0.047-0.255] | 0.114[0.042-0.206] | -0.008 | 0.014 | 0.582 | 0.868 |
| ***F. Oscillospiraceae-003*** | 0.335 ±0.370 | 0.506 ±0.623 | 0.239[0.072-0.458] | 0.305[0.113-0.759] | 0.041 | 0.011 | < 0.001 | 0.014 |
| ***G. Odoribacter*** | 0.541 ±1.575 | 0.338 ±0.326 | 0.278[0.109-0.495] | 0.257[0.141-0.424] | < 0.001 | 0.011 | 0.966 | 0.966 |
| ***G. Alistipes*** | 5.313 ±4.325 | 5.298 ±4.580 | 4.774[2.321-6.778] | 4.548[2.496-6.270] | 0.005 | 0.01 | 0.593 | 0.868 |
| ***G. Lachnospiraceae NK4A136 group*** | 1.280 ±1.040 | 1.726 ±1.602 | 1.050[0.452-1.726] | 1.263[0.692-2.028] | 0.029 | 0.009 | 0.001 | 0.03 |
| ***F. Oscillospiraceae-002*** | 2.785 ±1.901 | 2.901 ±2.318 | 2.495[1.559-3.535] | 2.437[1.336-3.841] | 0.007 | 0.009 | 0.445 | 0.868 |
| ***F. Oscillospiraceae-005*** | 0.723 ±0.635 | 0.705 ±0.631 | 0.579[0.303-0.936] | 0.554[0.224-1.104] | 0.001 | 0.009 | 0.885 | 0.966 |
| ***G. Escherichia-Shigella*** | 0.626 ±1.791 | 0.898 ±3.893 | 0.057[0.014-0.129] | 0.042[0.010-0.147] | 0.014 | NA | NA | NA |
| ***G. Blautia*** | 4.536 ±3.448 | 4.080 ±3.463 | 3.496[1.812-6.288] | 3.177[1.589-5.551] | -0.005 | 0.009 | 0.552 | 0.868 |
| ***G. Dorea*** | 1.338 ±1.052 | 1.235 ±1.381 | 1.122[0.695-1.594] | 0.766[0.394-1.618] | -0.009 | 0.011 | 0.407 | 0.868 |
| ***G. Enterorhabdus*** | 0.075 ±0.145 | 0.057 ±0.083 | 0.017[0.000-0.078] | 0.013[0.000-0.087] | -0.009 | 0.018 | 0.627 | 0.868 |
| ***G. Erysipelatoclostridium*** | 0.053 ±0.173 | 0.026 ±0.065 | 0.010[0.000-0.039] | 0.003[0.000-0.026] | -0.007 | NA | NA | NA |
| ***G. Erysipelotrichaceae UCG-003*** | 0.761 ±1.025 | 0.553 ±0.670 | 0.408[0.097-0.915] | 0.300[0.058-0.675] | -0.025 | 0.013 | 0.065 | 0.361 |
| ***F. Christensenellaceae*** | 0.030 ±0.038 | 0.018 ±0.030 | 0.018[0.000-0.042] | 0.010[0.000-0.022] | -0.042 | 0.013 | 0.001 | 0.03 |
| ***G. Bifidobacterium*** | 0.890 ±1.285 | 0.766 ±1.285 | 0.491[0.079-1.117] | 0.293[0.060-1.084] | -0.02 | 0.014 | 0.168 | 0.583 |
| ***G. Ruminococcus gauvreauii group*** | 0.234 ±0.309 | 0.211 ±0.303 | 0.113[0.015-0.326] | 0.096[0.000-0.264] | -0.001 | 0.013 | 0.927 | 0.966 |
| ***F. Eggerthellaceae*** | 0.279 ±0.386 | 0.200 ±0.340 | 0.108[0.000-0.444] | 0.049[0.000-0.234] | -0.024 | 0.014 | 0.095 | 0.493 |
| ***G. Ruminococcus torques group*** | 0.835 ±1.323 | 0.495 ±0.617 | 0.452[0.190-0.932] | 0.363[0.150-0.555] | -0.029 | 0.011 | 0.007 | 0.125 |
| ***G. Adlercreutzia*** | 0.126 ±0.239 | 0.087 ±0.160 | 0.000[0.000-0.143] | 0.015[0.000-0.092] | -0.036 | 0.019 | 0.053 | 0.361 |
| ***G. Romboutsia*** | 0.518 ±0.837 | 0.439 ±0.611 | 0.224[0.066-0.505] | 0.222[0.061-0.506] | -0.001 | 0.013 | 0.929 | 0.966 |
| ***G. Eubacterium ventriosum group*** | 0.237 ±0.390 | 0.170 ±0.216 | 0.122[0.065-0.246] | 0.102[0.045-0.229] | -0.014 | 0.012 | 0.255 | 0.732 |
| ***G. Collinsella*** | 0.415 ±0.595 | 0.292 ±0.471 | 0.167[0.029-0.676] | 0.121[0.029-0.316] | -0.016 | 0.011 | 0.145 | 0.555 |
| **LFHP-LIR** | | | | | | | | |
| ***G. Terrisporobacter*** | 0.088 ±0.292 | 0.168 ±0.427 | 0.000[0.000-0.014] | 0.000[0.000-0.039] | 0.092 | 0.03 | 0.002 | 0.065 |
| ***G. Turicibacter*** | 0.078 ±0.165 | 0.247 ±0.451 | 0.004[0.000-0.050] | 0.012[0.000-0.223] | 0.085 | 0.027 | 0.001 | 0.064 |
| ***G. Akkermansia*** | 1.816 ±3.309 | 2.022 ±4.338 | 0.224[0.040-2.549] | 0.470[0.045-1.616] | 0.011 | 0.029 | 0.69 | 0.836 |
| ***G. Muribaculaceae*** | 0.758 ±1.587 | 0.932 ±1.788 | 0.006[0.000-0.666] | 0.012[0.000-0.876] | 0.015 | 0.023 | 0.502 | 0.758 |
| ***G. Butyricimonas*** | 0.191 ±0.194 | 0.184 ±0.239 | 0.111[0.052-0.308] | 0.086[0.026-0.279] | -0.006 | 0.017 | 0.719 | 0.843 |
| ***F. Oscillospiraceae-003*** | 0.276 ±0.313 | 0.433 ±0.590 | 0.094[0.039-0.407] | 0.173[0.061-0.581] | 0.038 | 0.021 | 0.066 | 0.36 |
| ***G. Odoribacter*** | 0.438 ±0.629 | 0.288 ±0.253 | 0.304[0.190-0.461] | 0.169[0.092-0.494] | -0.019 | 0.016 | 0.218 | 0.56 |
| ***G. Alistipes*** | 4.645 ±4.317 | 4.952 ±4.600 | 3.786[1.687-6.105] | 3.548[1.875-6.264] | 0.01 | 0.012 | 0.388 | 0.696 |
| ***G. Lachnospiraceae NK4A136 group*** | 1.379 ±1.048 | 1.563 ±2.121 | 1.209[0.518-1.849] | 0.979[0.539-2.022] | 0.006 | 0.012 | 0.649 | 0.836 |
| ***F. Oscillospiraceae-002*** | 2.474 ±2.281 | 2.587 ±2.691 | 1.886[0.727-3.438] | 1.834[0.945-2.981] | 0.013 | 0.013 | 0.306 | 0.65 |
| ***F. Oscillospiraceae-005*** | 0.621 ±0.620 | 0.614 ±0.766 | 0.490[0.267-0.820] | 0.499[0.155-0.760] | -0.006 | 0.013 | 0.627 | 0.836 |
| ***G. Escherichia-Shigella*** | 0.373 ±1.344 | 0.621 ±1.603 | 0.030[0.001-0.196] | 0.028[0.006-0.179] | -0.021 | 0.002 | < 0.001 | < 0.001 |
| ***G. Blautia*** | 6.446 ±5.046 | 5.830 ±4.676 | 5.624[2.822-7.698] | 4.013[2.339-8.115] | -0.003 | 0.01 | 0.748 | 0.856 |
| ***G. Dorea*** | 1.283 ±1.005 | 1.299 ±1.018 | 1.032[0.656-1.554] | 0.986[0.634-1.545] | 0.008 | 0.011 | 0.468 | 0.758 |
| ***G. Enterorhabdus*** | 0.085 ±0.152 | 0.083 ±0.140 | 0.045[0.000-0.087] | 0.013[0.000-0.108] | 0.001 | 0.02 | 0.972 | 0.972 |
| ***G. Erysipelatoclostridium*** | 0.031 ±0.045 | 0.024 ±0.049 | 0.010[0.000-0.046] | 0.003[0.000-0.025] | -0.039 | 0.021 | 0.059 | 0.36 |
| ***G. Erysipelotrichaceae UCG-003*** | 0.662 ±1.038 | 0.708 ±1.157 | 0.355[0.092-0.791] | 0.313[0.141-0.576] | 0.012 | 0.018 | 0.514 | 0.763 |
| ***F. Christensenellaceae*** | 0.026 ±0.036 | 0.017 ±0.023 | 0.017[0.000-0.035] | 0.010[0.000-0.020] | -0.024 | 0.011 | 0.035 | 0.328 |
| ***G. Bifidobacterium*** | 0.881 ±1.157 | 0.665 ±0.879 | 0.351[0.182-1.217] | 0.324[0.084-0.858] | -0.029 | 0.018 | 0.108 | 0.441 |
| ***G. Ruminococcus gauvreauii group*** | 0.200 ±0.221 | 0.193 ±0.253 | 0.110[0.021-0.312] | 0.107[0.000-0.278] | -0.008 | 0.015 | 0.588 | 0.822 |
| ***F. Eggerthellaceae*** | 0.284 ±0.621 | 0.332 ±0.973 | 0.060[0.000-0.215] | 0.061[0.000-0.194] | -0.01 | 0.025 | 0.688 | 0.836 |
| ***G. Ruminococcus torques group*** | 0.796 ±0.761 | 0.567 ±0.500 | 0.661[0.283-0.947] | 0.449[0.213-0.789] | -0.02 | 0.014 | 0.156 | 0.478 |
| ***G. Adlercreutzia*** | 0.068 ±0.138 | 0.054 ±0.123 | 0.007[0.000-0.076] | 0.000[0.000-0.050] | -0.047 | 0.026 | 0.068 | 0.36 |
| ***G. Romboutsia*** | 0.481 ±0.489 | 0.386 ±0.773 | 0.303[0.071-0.783] | 0.158[0.056-0.350] | -0.032 | 0.017 | 0.063 | 0.36 |
| ***G. Eubacterium ventriosum group*** | 0.184 ±0.201 | 0.169 ±0.198 | 0.096[0.057-0.247] | 0.090[0.045-0.210] | -0.012 | 0.013 | 0.356 | 0.672 |
| ***G. Collinsella*** | 0.524 ±0.513 | 0.475 ±0.487 | 0.365[0.195-0.674] | 0.285[0.116-0.723] | -0.012 | 0.013 | 0.348 | 0.672 |
| **LFHP-MIR** | | | | | | | | |
| ***G. Terrisporobacter*** | 0.183 ±0.446 | 0.265 ±0.766 | 0.000[0.000-0.097] | 0.019[0.000-0.101] | 0.017 | 0.03 | 0.565 | 0.916 |
| ***G. Turicibacter*** | 0.111 ±0.211 | 0.264 ±0.534 | 0.000[0.000-0.123] | 0.020[0.000-0.187] | 0.09 | 0.036 | 0.012 | 0.14 |
| ***G. Akkermansia*** | 1.859 ±3.239 | 2.737 ±4.312 | 0.302[0.043-3.010] | 0.758[0.083-3.151] | 0.073 | 0.021 | 0.001 | 0.013 |
| ***G. Muribaculaceae*** | 0.811 ±1.929 | 0.987 ±2.029 | 0.000[0.000-0.321] | 0.035[0.000-1.205] | 0.067 | 0.033 | 0.039 | 0.272 |
| ***G. Butyricimonas*** | 0.142 ±0.175 | 0.129 ±0.166 | 0.060[0.000-0.254] | 0.056[0.000-0.187] | -0.001 | 0.016 | 0.928 | 0.992 |
| ***F. Oscillospiraceae-003*** | 0.338 ±0.533 | 0.376 ±0.614 | 0.105[0.036-0.353] | 0.203[0.056-0.456] | 0.019 | 0.016 | 0.22 | 0.668 |
| ***G. Odoribacter*** | 0.586 ±1.014 | 0.473 ±0.852 | 0.235[0.118-0.568] | 0.238[0.172-0.455] | -0.004 | 0.009 | 0.694 | 0.992 |
| ***G. Alistipes*** | 4.896 ±4.099 | 5.718 ±4.908 | 4.241[2.457-6.255] | 4.802[2.585-6.964] | 0.01 | 0.009 | 0.279 | 0.687 |
| ***G. Lachnospiraceae NK4A136 group*** | 1.350 ±1.189 | 1.411 ±1.436 | 0.996[0.389-1.919] | 1.071[0.496-1.815] | 0.003 | 0.01 | 0.769 | 0.992 |
| ***F. Oscillospiraceae-002*** | 2.611 ±2.672 | 3.099 ±2.779 | 2.157[0.820-3.392] | 2.202[1.119-3.997] | 0.021 | 0.012 | 0.068 | 0.345 |
| ***F. Oscillospiraceae-005*** | 0.636 ±0.626 | 0.651 ±0.686 | 0.471[0.179-0.907] | 0.515[0.157-0.815] | -0.002 | 0.011 | 0.849 | 0.992 |
| ***G. Escherichia-Shigella*** | 1.199 ±4.124 | 1.166 ±4.111 | 0.031[0.000-0.165] | 0.033[0.004-0.125] | -0.009 | NA | NA | NA |
| ***G. Blautia*** | 5.000 ±4.741 | 6.310 ±7.453 | 3.778[2.081-6.375] | 3.976[1.876-6.867] | 0.009 | 0.009 | 0.326 | 0.687 |
| ***G. Dorea*** | 1.388 ±1.159 | 1.289 ±1.063 | 1.064[0.525-1.829] | 1.085[0.522-1.793] | -0.005 | 0.009 | 0.567 | 0.916 |
| ***G. Enterorhabdus*** | 0.064 ±0.128 | 0.044 ±0.110 | 0.000[0.000-0.068] | 0.000[0.000-0.049] | -0.036 | 0.023 | 0.114 | 0.436 |
| ***G. Erysipelatoclostridium*** | 0.052 ±0.123 | 0.032 ±0.070 | 0.006[0.000-0.049] | 0.006[0.000-0.022] | -0.031 | 0.004 | < 0.001 | < 0.001 |
| ***G. Erysipelotrichaceae UCG-003*** | 0.668 ±1.115 | 0.723 ±1.148 | 0.211[0.105-0.704] | 0.348[0.147-0.762] | 0.022 | 0.012 | 0.053 | 0.318 |
| ***F. Christensenellaceae*** | 0.020 ±0.036 | 0.015 ±0.023 | 0.005[0.000-0.030] | 0.007[0.000-0.026] | -0.024 | 0.017 | 0.157 | 0.524 |
| ***G. Bifidobacterium*** | 1.213 ±1.180 | 0.756 ±0.863 | 0.891[0.364-1.558] | 0.443[0.169-1.081] | -0.043 | 0.012 | < 0.001 | 0.011 |
| ***G. Ruminococcus gauvreauii group*** | 0.227 ±0.364 | 0.212 ±0.378 | 0.121[0.014-0.268] | 0.085[0.000-0.177] | -0.015 | 0.014 | 0.284 | 0.687 |
| ***F. Eggerthellaceae*** | 0.242 ±0.377 | 0.163 ±0.209 | 0.036[0.000-0.394] | 0.054[0.000-0.292] | -0.036 | 0.016 | 0.026 | 0.213 |
| ***G. Ruminococcus torques group*** | 0.587 ±0.724 | 0.574 ±0.766 | 0.388[0.131-0.599] | 0.322[0.132-0.679] | 0.001 | 0.013 | 0.967 | 0.992 |
| ***G. Adlercreutzia*** | 0.116 ±0.257 | 0.105 ±0.235 | 0.000[0.000-0.067] | 0.001[0.000-0.071] | -0.002 | 0.013 | 0.855 | 0.992 |
| ***G. Romboutsia*** | 0.496 ±0.769 | 0.472 ±0.994 | 0.167[0.053-0.584] | 0.146[0.029-0.427] | -0.058 | 0.009 | < 0.001 | < 0.001 |
| ***G. Eubacterium ventriosum group*** | 0.291 ±0.413 | 0.181 ±0.286 | 0.090[0.040-0.377] | 0.094[0.025-0.210] | -0.059 | 0.014 | < 0.001 | 0.001 |
| ***G. Collinsella*** | 0.441 ±0.861 | 0.330 ±0.485 | 0.198[0.000-0.430] | 0.138[0.000-0.518] | -0.015 | 0.015 | 0.311 | 0.687 |

Intervention effects are indicated by the model coefficient and standard error of MaAsLin2 statistical models. Model coefficients represent the effect size, positive coefficients indicate an increased relative abundance of a specific genus. Only significant, diet-induced changes with FDR adjusted p-value (q-values) equal or less than 0.0.5 in one or more phenotype x diet combinations being statistically significant. FDR, false discovery rate; HMUFA, high mono-unsaturated fatty acid diet; LFHP, low fat, high protein, high fiber diet; LIR, liver insulin resistance; MIR, muscle insulin resistance.

**Supplementary Table 9. Changes in individual fecal microbial genus abundances after a 12-week HMUFA or LFHP diet that were significant in tissue-specific LIR or MIR phenotypes, related to Figure 3. Prevalence cutoff >25%.**

| **Taxa** | **Mean ±SD(%)** | **Mean ±SD(%)** | **Median[IQR:25%-75%](%)** | **Median[IQR:25%-75%](%)** | **Coef** | **SE** | **P-value** | **Q-value** |
| --- | --- | --- | --- | --- | --- | --- | --- | --- |
|  | **{Week 0}** | **{Week 12}** | **{Week 0}** | **{Week 12}** |  |  |  |  |
| **HMUFA-LIR** | | | | | | | | |
| ***G. Alloprevotella*** | 0.152 ±0.723 | 0.188 ±0.950 | 0.000[0.000-0.000] | 0.000[0.000-0.007] | 0.282 | 0.005 | < 0.001 | < 0.001 |
| ***G. Enterobacter*** | 0.089 ±0.417 | 0.039 ±0.160 | 0.000[0.000-0.009] | 0.000[0.000-0.002] | -0.046 | 0.041 | 0.268 | 0.425 |
| ***O. Bacteroidales*** | 0.154 ±0.388 | 0.106 ±0.268 | 0.000[0.000-0.000] | 0.000[0.000-0.006] | -0.06 | 0.031 | 0.054 | 0.164 |
| ***G. Butyrivibrio*** | 0.138 ±0.431 | 0.171 ±0.586 | 0.000[0.000-0.005] | 0.000[0.000-0.008] | 0.006 | 0.038 | 0.876 | 0.946 |
| ***G. Turicibacter*** | 0.100 ±0.175 | 0.105 ±0.165 | 0.008[0.000-0.119] | 0.032[0.004-0.091] | -0.006 | 0.006 | 0.322 | 0.474 |
| ***G. Succinivibrio*** | 0.020 ±0.045 | 0.027 ±0.067 | 0.000[0.000-0.013] | 0.000[0.000-0.017] | 0.087 | 0.013 | < 0.001 | < 0.001 |
| ***G. Intestinimonas*** | 0.015 ±0.062 | 0.004 ±0.009 | 0.000[0.000-0.008] | 0.000[0.000-0.005] | -0.059 | 0.026 | 0.022 | 0.093 |
| ***G. Lachnospiraceae UCG-003*** | 0.002 ±0.008 | 0.021 ±0.065 | 0.000[0.000-0.000] | 0.000[0.000-0.000] | 0.084 | 0.028 | 0.003 | 0.021 |
| ***G. Muribaculaceae*** | 0.938 ±2.863 | 0.886 ±2.777 | 0.000[0.000-0.301] | 0.006[0.000-0.761] | 0.042 | 0.024 | 0.077 | 0.203 |
| ***G. Methanobrevibacter*** | 0.169 ±0.515 | 0.503 ±1.758 | 0.016[0.000-0.088] | 0.016[0.000-0.118] | 0.064 | 0.027 | 0.019 | 0.086 |
| ***G. Oscillospira*** | 0.010 ±0.026 | 0.029 ±0.123 | 0.000[0.000-0.000] | 0.000[0.000-0.011] | 0.028 | NA | NA | NA |
| ***G. Christensenella*** | 0.003 ±0.007 | 0.004 ±0.007 | 0.000[0.000-0.005] | 0.000[0.000-0.006] | 0.021 | 0.023 | 0.362 | 0.503 |
| ***G. Butyricimonas*** | 0.157 ±0.165 | 0.255 ±0.282 | 0.108[0.033-0.232] | 0.152[0.043-0.353] | 0.046 | 0.015 | 0.002 | 0.017 |
| ***G. Flavonifractor*** | 0.036 ±0.081 | 0.023 ±0.064 | 0.000[0.000-0.024] | 0.000[0.000-0.008] | -0.034 | NA | NA | NA |
| ***G. Eubacterium xylanophilum group*** | 0.214 ±0.221 | 0.323 ±0.332 | 0.157[0.034-0.314] | 0.244[0.124-0.418] | 0.04 | 0.016 | 0.016 | 0.078 |
| ***G. Eubacterium siraeum group*** | 0.455 ±0.628 | 0.814 ±1.359 | 0.222[0.018-0.737] | 0.099[0.042-0.718] | 0.033 | 0.014 | 0.022 | 0.093 |
| ***G. Odoribacter*** | 0.329 ±0.344 | 0.515 ±0.760 | 0.179[0.102-0.404] | 0.271[0.121-0.543] | 0.038 | 0.013 | 0.003 | 0.023 |
| ***F. Oscillospiraceae-003*** | 0.337 ±0.449 | 0.433 ±0.355 | 0.180[0.081-0.514] | 0.346[0.126-0.627] | 0.037 | 0.015 | 0.012 | 0.07 |
| ***G. Paraprevotella*** | 0.498 ±0.816 | 0.597 ±0.950 | 0.161[0.000-0.686] | 0.139[0.000-0.736] | 0.034 | 0.015 | 0.023 | 0.093 |
| ***G. Alistipes*** | 4.950 ±3.852 | 6.361 ±4.413 | 4.399[2.097-6.989] | 5.268[3.210-7.590] | 0.033 | 0.008 | < 0.001 | 0.001 |
| ***G. Lachnospiraceae NK4A136 group*** | 2.090 ±2.500 | 2.540 ±2.303 | 1.274[0.762-2.265] | 2.500[0.767-3.804] | 0.032 | 0.015 | 0.027 | 0.104 |
| ***F. Oscillospiraceae-002*** | 2.425 ±1.970 | 3.072 ±2.441 | 2.210[0.834-3.146] | 2.728[1.397-4.100] | 0.029 | 0.009 | 0.001 | 0.012 |
| ***G. Barnesiella*** | 1.357 ±1.688 | 1.737 ±2.065 | 0.739[0.305-1.760] | 0.850[0.424-2.496] | 0.027 | 0.012 | 0.019 | 0.086 |
| ***G. Asteroleplasma*** | 0.004 ±0.022 | 0.017 ±0.090 | 0.000[0.000-0.000] | 0.000[0.000-0.000] | 0.024 | 0.008 | 0.002 | 0.017 |
| ***G. Rikenella*** | 0.005 ±0.021 | 0.005 ±0.012 | 0.000[0.000-0.000] | 0.000[0.000-0.000] | 0.018 | 0.005 | 0.001 | 0.008 |
| ***F. Oscillospiraceae-005*** | 0.732 ±0.891 | 0.861 ±1.149 | 0.435[0.246-0.906] | 0.601[0.251-1.071] | 0.023 | 0.008 | 0.005 | 0.037 |
| ***O. Coriobacteriales g1*** | 0.116 ±0.319 | 0.068 ±0.125 | 0.021[0.000-0.082] | 0.004[0.000-0.085] | -0.049 | NA | NA | NA |
| ***G. Allisonella*** | 0.024 ±0.044 | 0.014 ±0.028 | 0.000[0.000-0.029] | 0.000[0.000-0.020] | -0.027 | 0.011 | 0.015 | 0.076 |
| ***G. Blautia*** | 5.912 ±4.891 | 4.270 ±4.601 | 4.011[2.734-6.762] | 2.684[1.379-5.788] | -0.029 | 0.009 | 0.001 | 0.013 |
| ***G. Dorea*** | 1.528 ±1.241 | 1.005 ±0.895 | 1.192[0.645-1.731] | 0.693[0.335-1.357] | -0.032 | 0.01 | 0.002 | 0.017 |
| ***F. Lachnospiraceae-900066575*** | 0.024 ±0.030 | 0.025 ±0.026 | 0.015[0.000-0.038] | 0.022[0.000-0.034] | 0.005 | 0.012 | 0.671 | 0.781 |
| ***G. Erysipelotrichaceae UCG-003*** | 0.842 ±1.103 | 0.511 ±0.640 | 0.458[0.216-0.941] | 0.311[0.080-0.640] | -0.038 | 0.013 | 0.002 | 0.02 |
| ***G. Romboutsia*** | 0.581 ±0.622 | 0.391 ±0.483 | 0.350[0.116-0.898] | 0.167[0.042-0.654] | -0.039 | 0.016 | 0.014 | 0.076 |
| ***G. Escherichia-Shigella*** | 0.661 ±2.040 | 0.399 ±1.483 | 0.047[0.008-0.138] | 0.046[0.007-0.078] | -0.041 | 0.018 | 0.025 | 0.1 |
| ***G. Lactobacillus*** | 0.514 ±2.057 | 0.052 ±0.172 | 0.012[0.000-0.061] | 0.000[0.000-0.020] | -0.029 | NA | NA | NA |
| ***F. Butyricicoccaceae-009*** | 0.016 ±0.044 | 0.013 ±0.027 | 0.000[0.000-0.012] | 0.002[0.000-0.017] | 0.002 | 0.019 | 0.908 | 0.948 |
| ***G. Ruminococcus gauvreauii group*** | 0.313 ±0.387 | 0.157 ±0.159 | 0.195[0.089-0.353] | 0.128[0.031-0.204] | -0.046 | 0.012 | < 0.001 | 0.003 |
| ***G. Ruminococcus torques group*** | 0.604 ±0.485 | 0.336 ±0.352 | 0.458[0.248-0.965] | 0.203[0.115-0.398] | -0.046 | 0.012 | < 0.001 | 0.002 |
| ***F. Christensenellaceae*** | 0.016 ±0.023 | 0.019 ±0.024 | 0.007[0.000-0.022] | 0.010[0.000-0.026] | 0.031 | 0.019 | 0.098 | 0.235 |
| ***G. Enterorhabdus*** | 0.092 ±0.215 | 0.050 ±0.103 | 0.038[0.000-0.085] | 0.015[0.000-0.067] | -0.038 | 0.014 | 0.006 | 0.038 |
| ***F. Eggerthellaceae*** | 0.260 ±0.638 | 0.118 ±0.276 | 0.014[0.000-0.197] | 0.014[0.000-0.106] | -0.05 | 0.019 | 0.008 | 0.051 |
| ***G. Adlercreutzia*** | 0.130 ±0.307 | 0.090 ±0.186 | 0.000[0.000-0.102] | 0.000[0.000-0.039] | -0.061 | 0.021 | 0.003 | 0.023 |
| ***G. Ruminococcus gnavus group*** | 0.021 ±0.065 | 0.013 ±0.072 | 0.000[0.000-0.000] | 0.000[0.000-0.000] | 0.085 | NA | NA | NA |
| ***G. Collinsella*** | 0.495 ±0.498 | 0.276 ±0.378 | 0.370[0.161-0.657] | 0.155[0.082-0.307] | -0.063 | 0.011 | < 0.001 | < 0.001 |
| ***F. Prevotellaceae*** | 0.025 ±0.098 | 0.042 ±0.164 | 0.000[0.000-0.000] | 0.000[0.000-0.000] | -0.075 | 0.005 | < 0.001 | < 0.001 |
| ***G. Catenibacterium*** | 0.508 ±1.576 | 0.123 ±0.443 | 0.000[0.000-0.046] | 0.000[0.000-0.003] | -0.076 | 0.023 | 0.001 | 0.012 |
| ***P. Firmicutes-DTU014*** | 0.018 ±0.052 | 0.028 ±0.052 | 0.000[0.000-0.005] | 0.000[0.000-0.043] | 0.034 | 0.022 | 0.13 | 0.278 |
| ***G. Eisenbergiella*** | 0.011 ±0.030 | 0.002 ±0.004 | 0.000[0.000-0.000] | 0.000[0.000-0.000] | -0.057 | 0.024 | 0.017 | 0.084 |
| ***G. Olsenella*** | 0.013 ±0.033 | 0.006 ±0.023 | 0.000[0.000-0.003] | 0.000[0.000-0.000] | -0.089 | 0.035 | 0.011 | 0.066 |
| ***G. Lactococcus*** | 0.039 ±0.108 | 0.001 ±0.002 | 0.000[0.000-0.022] | 0.000[0.000-0.000] | -0.35 | 0.064 | < 0.001 | < 0.001 |
| **HMUFA-MIR** | | | | | | | | |
| ***G. Alloprevotella*** | 0.396 ±1.045 | 0.526 ±1.421 | 0.000[0.000-0.002] | 0.000[0.000-0.003] | -0.013 | 0.035 | 0.711 | 0.892 |
| ***G. Enterobacter*** | 0.129 ±0.889 | 0.019 ±0.089 | 0.000[0.000-0.000] | 0.000[0.000-0.000] | -0.064 | 0.017 | < 0.001 | 0.003 |
| ***O. Bacteroidales*** | 0.068 ±0.222 | 0.037 ±0.115 | 0.000[0.000-0.000] | 0.000[0.000-0.000] | 0.215 | 0.015 | < 0.001 | < 0.001 |
| ***G. Butyrivibrio*** | 0.058 ±0.272 | 0.108 ±0.403 | 0.000[0.000-0.000] | 0.000[0.000-0.000] | 0.027 | 0.012 | 0.03 | 0.174 |
| ***G. Turicibacter*** | 0.125 ±0.259 | 0.115 ±0.247 | 0.012[0.000-0.068] | 0.018[0.000-0.085] | -0.001 | 0.024 | 0.957 | 0.987 |
| ***G. Succinivibrio*** | 0.015 ±0.061 | 0.091 ±0.608 | 0.000[0.000-0.000] | 0.000[0.000-0.006] | -0.06 | 0.008 | < 0.001 | < 0.001 |
| ***G. Intestinimonas*** | 0.018 ±0.078 | 0.006 ±0.017 | 0.000[0.000-0.004] | 0.000[0.000-0.000] | -0.013 | 0.018 | 0.45 | 0.842 |
| ***G. Lachnospiraceae UCG-003*** | 0.009 ±0.037 | 0.011 ±0.035 | 0.000[0.000-0.000] | 0.000[0.000-0.000] | 0.02 | 0.036 | 0.581 | 0.843 |
| ***G. Muribaculaceae*** | 0.734 ±2.024 | 1.149 ±2.445 | 0.004[0.000-0.656] | 0.004[0.000-0.991] | 0.063 | 0.018 | 0.001 | 0.009 |
| ***G. Methanobrevibacter*** | 0.705 ±1.803 | 0.336 ±0.797 | 0.065[0.005-0.300] | 0.037[0.004-0.174] | -0.001 | NA | NA | NA |
| ***G. Oscillospira*** | 0.047 ±0.244 | 0.021 ±0.084 | 0.000[0.000-0.011] | 0.000[0.000-0.010] | -0.006 | 0.026 | 0.813 | 0.924 |
| ***G. Christensenella*** | 0.005 ±0.009 | 0.004 ±0.009 | 0.000[0.000-0.003] | 0.000[0.000-0.006] | -0.019 | 0.019 | 0.317 | 0.675 |
| ***G. Butyricimonas*** | 0.199 ±0.216 | 0.167 ±0.202 | 0.133[0.047-0.255] | 0.114[0.042-0.206] | -0.015 | 0.014 | 0.289 | 0.645 |
| ***G. Flavonifractor*** | 0.040 ±0.101 | 0.028 ±0.071 | 0.000[0.000-0.030] | 0.000[0.000-0.027] | -0.027 | 0.014 | 0.058 | 0.257 |
| ***G. Eubacterium xylanophilum group*** | 0.218 ±0.240 | 0.265 ±0.293 | 0.150[0.040-0.342] | 0.186[0.056-0.326] | 0.014 | 0.012 | 0.233 | 0.589 |
| ***G. Eubacterium siraeum group*** | 0.637 ±1.061 | 0.636 ±1.321 | 0.258[0.029-0.655] | 0.200[0.019-0.591] | -0.012 | 0.02 | 0.536 | 0.842 |
| ***G. Odoribacter*** | 0.541 ±1.575 | 0.338 ±0.326 | 0.278[0.109-0.495] | 0.257[0.141-0.424] | -0.006 | 0.01 | 0.54 | 0.842 |
| ***F. Oscillospiraceae-003*** | 0.335 ±0.370 | 0.506 ±0.623 | 0.239[0.072-0.458] | 0.305[0.113-0.759] | 0.034 | 0.012 | 0.004 | 0.047 |
| ***G. Paraprevotella*** | 0.442 ±0.709 | 0.404 ±0.617 | 0.106[0.006-0.542] | 0.129[0.000-0.493] | 0.005 | 0.016 | 0.752 | 0.905 |
| ***G. Alistipes*** | 5.313 ±4.325 | 5.298 ±4.580 | 4.774[2.321-6.778] | 4.548[2.496-6.270] | < 0.001 | 0.009 | 0.999 | 0.999 |
| ***G. Lachnospiraceae NK4A136 group*** | 1.280 ±1.040 | 1.726 ±1.602 | 1.050[0.452-1.726] | 1.263[0.692-2.028] | 0.022 | 0.008 | 0.005 | 0.058 |
| ***F. Oscillospiraceae-002*** | 2.785 ±1.901 | 2.901 ±2.318 | 2.495[1.559-3.535] | 2.437[1.336-3.841] | < 0.001 | 0.009 | 0.978 | 0.987 |
| ***G. Barnesiella*** | 1.131 ±1.246 | 1.219 ±1.356 | 0.650[0.182-1.609] | 0.673[0.121-2.027] | 0.005 | 0.014 | 0.705 | 0.892 |
| ***G. Asteroleplasma*** | 0.098 ±0.636 | 0.105 ±0.749 | 0.000[0.000-0.000] | 0.000[0.000-0.000] | 0.057 | NA | NA | NA |
| ***G. Rikenella*** | 0.002 ±0.007 | 0.003 ±0.010 | 0.000[0.000-0.000] | 0.000[0.000-0.000] | -0.034 | NA | NA | NA |
| ***F. Oscillospiraceae-005*** | 0.723 ±0.635 | 0.705 ±0.631 | 0.579[0.303-0.936] | 0.554[0.224-1.104] | -0.004 | 0.009 | 0.685 | 0.892 |
| ***O. Coriobacteriales g1*** | 0.140 ±0.468 | 0.129 ±0.433 | 0.000[0.000-0.063] | 0.005[0.000-0.032] | -0.014 | 0.023 | 0.561 | 0.842 |
| ***G. Allisonella*** | 0.016 ±0.040 | 0.021 ±0.057 | 0.000[0.000-0.007] | 0.000[0.000-0.018] | 0.008 | 0.028 | 0.784 | 0.905 |
| ***G. Blautia*** | 4.536 ±3.448 | 4.080 ±3.463 | 3.496[1.812-6.288] | 3.177[1.589-5.551] | -0.012 | 0.008 | 0.138 | 0.41 |
| ***G. Dorea*** | 1.338 ±1.052 | 1.235 ±1.381 | 1.122[0.695-1.594] | 0.766[0.394-1.618] | -0.018 | 0.01 | 0.071 | 0.271 |
| ***F. Lachnospiraceae-900066575*** | 0.023 ±0.031 | 0.019 ±0.026 | 0.011[0.000-0.035] | 0.012[0.000-0.030] | -0.009 | 0.012 | 0.436 | 0.835 |
| ***G. Erysipelotrichaceae UCG-003*** | 0.761 ±1.025 | 0.553 ±0.670 | 0.408[0.097-0.915] | 0.300[0.058-0.675] | -0.031 | 0.013 | 0.021 | 0.173 |
| ***G. Romboutsia*** | 0.518 ±0.837 | 0.439 ±0.611 | 0.224[0.066-0.505] | 0.222[0.061-0.506] | -0.007 | 0.013 | 0.561 | 0.842 |
| ***G. Escherichia-Shigella*** | 0.626 ±1.791 | 0.898 ±3.893 | 0.057[0.014-0.129] | 0.042[0.010-0.147] | -0.031 | 0.022 | 0.164 | 0.459 |
| ***G. Lactobacillus*** | 0.204 ±0.657 | 0.033 ±0.094 | 0.000[0.000-0.089] | 0.000[0.000-0.012] | -0.045 | 0.01 | < 0.001 | < 0.001 |
| ***F. Butyricicoccaceae-009*** | 0.020 ±0.030 | 0.013 ±0.017 | 0.012[0.000-0.026] | 0.006[0.000-0.019] | -0.045 | 0.016 | 0.004 | 0.047 |
| ***G. Ruminococcus gauvreauii group*** | 0.234 ±0.309 | 0.211 ±0.303 | 0.113[0.015-0.326] | 0.096[0.000-0.264] | -0.008 | 0.012 | 0.526 | 0.842 |
| ***G. Ruminococcus torques group*** | 0.835 ±1.323 | 0.495 ±0.617 | 0.452[0.190-0.932] | 0.363[0.150-0.555] | -0.034 | 0.01 | 0.001 | 0.012 |
| ***F. Christensenellaceae*** | 0.030 ±0.038 | 0.018 ±0.030 | 0.018[0.000-0.042] | 0.010[0.000-0.022] | -0.046 | 0.013 | 0.001 | 0.009 |
| ***G. Enterorhabdus*** | 0.075 ±0.145 | 0.057 ±0.083 | 0.017[0.000-0.078] | 0.013[0.000-0.087] | -0.015 | 0.018 | 0.396 | 0.781 |
| ***F. Eggerthellaceae*** | 0.279 ±0.386 | 0.200 ±0.340 | 0.108[0.000-0.444] | 0.049[0.000-0.234] | -0.03 | 0.014 | 0.026 | 0.174 |
| ***G. Adlercreutzia*** | 0.126 ±0.239 | 0.087 ±0.160 | 0.000[0.000-0.143] | 0.015[0.000-0.092] | -0.043 | 0.018 | 0.019 | 0.168 |
| ***G. Ruminococcus gnavus group*** | 0.020 ±0.046 | 0.013 ±0.035 | 0.000[0.000-0.008] | 0.000[0.000-0.000] | -0.062 | 0.012 | < 0.001 | < 0.001 |
| ***G. Collinsella*** | 0.415 ±0.595 | 0.292 ±0.471 | 0.167[0.029-0.676] | 0.121[0.029-0.316] | -0.025 | 0.01 | 0.016 | 0.154 |
| ***F. Prevotellaceae*** | 0.176 ±0.426 | 0.141 ±0.351 | 0.000[0.000-0.185] | 0.000[0.000-0.101] | 0.01 | 0.017 | 0.535 | 0.842 |
| ***G. Catenibacterium*** | 0.321 ±0.791 | 0.398 ±1.159 | 0.000[0.000-0.074] | 0.000[0.000-0.005] | -0.013 | 0.029 | 0.641 | 0.867 |
| ***P. Firmicutes-DTU014*** | 0.027 ±0.053 | 0.021 ±0.042 | 0.003[0.000-0.030] | 0.000[0.000-0.025] | -0.005 | 0.019 | 0.783 | 0.905 |
| ***G. Eisenbergiella*** | 0.025 ±0.098 | 0.011 ±0.053 | 0.000[0.000-0.000] | 0.000[0.000-0.000] | -0.089 | 0.043 | 0.039 | 0.207 |
| ***G. Olsenella*** | 0.014 ±0.043 | 0.010 ±0.023 | 0.000[0.000-0.000] | 0.000[0.000-0.000] | -0.05 | NA | NA | NA |
| ***G. Lactococcus*** | 0.159 ±0.717 | 0.003 ±0.008 | 0.000[0.000-0.027] | 0.000[0.000-0.000] | -0.075 | 0.003 | < 0.001 | < 0.001 |
| **LFHP-LIR** | | | | | | | | |
| ***G. Alloprevotella*** | 0.532 ±1.330 | 0.399 ±0.932 | 0.000[0.000-0.005] | 0.000[0.000-0.007] | -0.021 | 0.03 | 0.485 | 0.716 |
| ***G. Enterobacter*** | 0.272 ±1.686 | 0.184 ±0.693 | 0.000[0.000-0.000] | 0.000[0.000-0.007] | 0.253 | 0.013 | < 0.001 | < 0.001 |
| ***O. Bacteroidales*** | 0.054 ±0.144 | 0.097 ±0.362 | 0.000[0.000-0.001] | 0.000[0.000-0.000] | 0.123 | NA | NA | NA |
| ***G. Butyrivibrio*** | 0.233 ±0.826 | 0.552 ±1.491 | 0.000[0.000-0.073] | 0.000[0.000-0.138] | 0.118 | 0.041 | 0.004 | 0.048 |
| ***G. Turicibacter*** | 0.078 ±0.165 | 0.247 ±0.451 | 0.004[0.000-0.050] | 0.012[0.000-0.223] | 0.088 | 0.03 | 0.003 | 0.037 |
| ***G. Succinivibrio*** | 0.064 ±0.317 | 0.050 ±0.276 | 0.000[0.000-0.003] | 0.000[0.000-0.003] | -0.027 | 0.034 | 0.429 | 0.68 |
| ***G. Intestinimonas*** | 0.006 ±0.013 | 0.015 ±0.030 | 0.000[0.000-0.009] | 0.000[0.000-0.021] | 0.086 | 0.021 | < 0.001 | 0.001 |
| ***G. Lachnospiraceae UCG-003*** | 0.007 ±0.024 | 0.018 ±0.084 | 0.000[0.000-0.000] | 0.000[0.000-0.000] | -0.029 | 0.02 | 0.152 | 0.48 |
| ***G. Muribaculaceae*** | 0.758 ±1.587 | 0.932 ±1.788 | 0.006[0.000-0.666] | 0.012[0.000-0.876] | 0.01 | 0.023 | 0.648 | 0.847 |
| ***G. Methanobrevibacter*** | 0.327 ±1.011 | 0.696 ±2.510 | 0.021[0.000-0.089] | 0.005[0.000-0.163] | 0.01 | 0.027 | 0.723 | 0.896 |
| ***G. Oscillospira*** | 0.012 ±0.035 | 0.019 ±0.080 | 0.000[0.000-0.009] | 0.000[0.000-0.009] | 0.062 | 0.006 | < 0.001 | < 0.001 |
| ***G. Christensenella*** | 0.002 ±0.006 | 0.004 ±0.009 | 0.000[0.000-0.000] | 0.000[0.000-0.000] | 0.052 | 0.017 | 0.002 | 0.033 |
| ***G. Butyricimonas*** | 0.191 ±0.194 | 0.184 ±0.239 | 0.111[0.052-0.308] | 0.086[0.026-0.279] | -0.004 | 0.017 | 0.813 | 0.906 |
| ***G. Flavonifractor*** | 0.020 ±0.057 | 0.033 ±0.067 | 0.000[0.000-0.012] | 0.011[0.000-0.037] | 0.046 | 0.016 | 0.005 | 0.056 |
| ***G. Eubacterium xylanophilum group*** | 0.215 ±0.316 | 0.196 ±0.290 | 0.095[0.024-0.267] | 0.090[0.015-0.265] | < 0.001 | 0.019 | 0.979 | 0.994 |
| ***G. Eubacterium siraeum group*** | 0.533 ±0.651 | 0.582 ±0.943 | 0.218[0.056-0.803] | 0.181[0.014-0.698] | 0.04 | 0.027 | 0.148 | 0.48 |
| ***G. Odoribacter*** | 0.438 ±0.629 | 0.288 ±0.253 | 0.304[0.190-0.461] | 0.169[0.092-0.494] | -0.021 | 0.016 | 0.176 | 0.492 |
| ***F. Oscillospiraceae-003*** | 0.276 ±0.313 | 0.433 ±0.590 | 0.094[0.039-0.407] | 0.173[0.061-0.581] | 0.06 | NA | NA | NA |
| ***G. Paraprevotella*** | 0.576 ±0.967 | 0.561 ±0.789 | 0.216[0.021-0.566] | 0.174[0.022-1.019] | -0.003 | 0.018 | 0.852 | 0.908 |
| ***G. Alistipes*** | 4.645 ±4.317 | 4.952 ±4.600 | 3.786[1.687-6.105] | 3.548[1.875-6.264] | 0.01 | 0.012 | 0.417 | 0.68 |
| ***G. Lachnospiraceae NK4A136 group*** | 1.379 ±1.048 | 1.563 ±2.121 | 1.209[0.518-1.849] | 0.979[0.539-2.022] | 0.004 | 0.013 | 0.773 | 0.898 |
| ***F. Oscillospiraceae-002*** | 2.474 ±2.281 | 2.587 ±2.691 | 1.886[0.727-3.438] | 1.834[0.945-2.981] | 0.012 | 0.012 | 0.344 | 0.668 |
| ***G. Barnesiella*** | 1.335 ±1.382 | 1.418 ±2.012 | 0.871[0.234-2.029] | 0.564[0.144-1.495] | -0.009 | 0.017 | 0.6 | 0.841 |
| ***G. Asteroleplasma*** | 0.014 ±0.040 | 0.025 ±0.063 | 0.000[0.000-0.003] | 0.000[0.000-0.005] | -0.022 | 0.005 | < 0.001 | < 0.001 |
| ***G. Rikenella*** | 0.004 ±0.012 | 0.001 ±0.004 | 0.000[0.000-0.000] | 0.000[0.000-0.000] | 0.024 | 0.031 | 0.435 | 0.68 |
| ***F. Oscillospiraceae-005*** | 0.621 ±0.620 | 0.614 ±0.766 | 0.490[0.267-0.820] | 0.499[0.155-0.760] | -0.008 | 0.013 | 0.52 | 0.745 |
| ***O. Coriobacteriales g1*** | 0.077 ±0.162 | 0.086 ±0.157 | 0.012[0.000-0.066] | 0.015[0.000-0.081] | 0.02 | 0.006 | 0.001 | 0.033 |
| ***G. Allisonella*** | 0.026 ±0.062 | 0.019 ±0.052 | 0.000[0.000-0.020] | 0.000[0.000-0.007] | -0.008 | 0.025 | 0.74 | 0.898 |
| ***G. Blautia*** | 6.446 ±5.046 | 5.830 ±4.676 | 5.624[2.822-7.698] | 4.013[2.339-8.115] | -0.004 | 0.009 | 0.63 | 0.847 |
| ***G. Dorea*** | 1.283 ±1.005 | 1.299 ±1.018 | 1.032[0.656-1.554] | 0.986[0.634-1.545] | 0.009 | 0.01 | 0.374 | 0.68 |
| ***F. Lachnospiraceae-900066575*** | 0.025 ±0.024 | 0.017 ±0.016 | 0.019[0.000-0.041] | 0.013[0.000-0.023] | -0.032 | 0.01 | 0.002 | 0.034 |
| ***G. Erysipelotrichaceae UCG-003*** | 0.662 ±1.038 | 0.708 ±1.157 | 0.355[0.092-0.791] | 0.313[0.141-0.576] | 0.012 | 0.016 | 0.458 | 0.692 |
| ***G. Romboutsia*** | 0.481 ±0.489 | 0.386 ±0.773 | 0.303[0.071-0.783] | 0.158[0.056-0.350] | -0.03 | 0.017 | 0.081 | 0.406 |
| ***G. Escherichia-Shigella*** | 0.373 ±1.344 | 0.621 ±1.603 | 0.030[0.001-0.196] | 0.028[0.006-0.179] | -0.009 | 0.005 | 0.08 | 0.406 |
| ***G. Lactobacillus*** | 0.278 ±1.322 | 0.124 ±0.450 | 0.000[0.000-0.057] | 0.000[0.000-0.030] | 0.005 | 0.037 | 0.892 | 0.933 |
| ***F. Butyricicoccaceae-009*** | 0.030 ±0.077 | 0.086 ±0.412 | 0.007[0.000-0.026] | 0.002[0.000-0.018] | 0.008 | NA | NA | NA |
| ***G. Ruminococcus gauvreauii group*** | 0.200 ±0.221 | 0.193 ±0.253 | 0.110[0.021-0.312] | 0.107[0.000-0.278] | -0.003 | 0.013 | 0.82 | 0.906 |
| ***G. Ruminococcus torques group*** | 0.796 ±0.761 | 0.567 ±0.500 | 0.661[0.283-0.947] | 0.449[0.213-0.789] | -0.019 | 0.014 | 0.175 | 0.492 |
| ***F. Christensenellaceae*** | 0.026 ±0.036 | 0.017 ±0.023 | 0.017[0.000-0.035] | 0.010[0.000-0.020] | -0.028 | 0.013 | 0.036 | 0.269 |
| ***G. Enterorhabdus*** | 0.085 ±0.152 | 0.083 ±0.140 | 0.045[0.000-0.087] | 0.013[0.000-0.108] | < 0.001 | 0.019 | 0.996 | 0.996 |
| ***F. Eggerthellaceae*** | 0.284 ±0.621 | 0.332 ±0.973 | 0.060[0.000-0.215] | 0.061[0.000-0.194] | -0.009 | 0.024 | 0.706 | 0.888 |
| ***G. Adlercreutzia*** | 0.068 ±0.138 | 0.054 ±0.123 | 0.007[0.000-0.076] | 0.000[0.000-0.050] | -0.034 | 0.024 | 0.163 | 0.492 |
| ***G. Ruminococcus gnavus group*** | 0.029 ±0.084 | 0.076 ±0.372 | 0.000[0.000-0.013] | 0.000[0.000-0.000] | -0.05 | 0.06 | 0.408 | 0.68 |
| ***G. Collinsella*** | 0.524 ±0.513 | 0.475 ±0.487 | 0.365[0.195-0.674] | 0.285[0.116-0.723] | -0.011 | 0.013 | 0.419 | 0.68 |
| ***F. Prevotellaceae*** | 0.084 ±0.212 | 0.085 ±0.226 | 0.000[0.000-0.007] | 0.000[0.000-0.005] | -0.01 | NA | NA | NA |
| ***G. Catenibacterium*** | 0.612 ±1.736 | 0.671 ±1.701 | 0.000[0.000-0.078] | 0.000[0.000-0.020] | 0.044 | 0.02 | 0.024 | 0.232 |
| ***P. Firmicutes-DTU014*** | 0.018 ±0.031 | 0.009 ±0.023 | 0.000[0.000-0.022] | 0.000[0.000-0.008] | -0.078 | 0.022 | < 0.001 | 0.009 |
| ***G. Eisenbergiella*** | 0.002 ±0.005 | 0.005 ±0.012 | 0.000[0.000-0.000] | 0.000[0.000-0.000] | 0.075 | 0.024 | 0.002 | 0.033 |
| ***G. Olsenella*** | 0.023 ±0.051 | 0.023 ±0.065 | 0.000[0.000-0.020] | 0.000[0.000-0.012] | 0.027 | 0.034 | 0.433 | 0.68 |
| ***G. Lactococcus*** | 0.014 ±0.030 | 0.010 ±0.021 | 0.000[0.000-0.015] | 0.000[0.000-0.006] | -0.023 | 0.029 | 0.433 | 0.68 |
| **LFHP-MIR** | | | | | | | | |
| ***G. Alloprevotella*** | 0.457 ±1.907 | 0.254 ±0.769 | 0.000[0.000-0.000] | 0.000[0.000-0.001] | 0.005 | 0.05 | 0.927 | 0.995 |
| ***G. Enterobacter*** | 0.064 ±0.217 | 0.041 ±0.149 | 0.000[0.000-0.026] | 0.000[0.000-0.001] | 0.011 | NA | NA | NA |
| ***O. Bacteroidales*** | 0.149 ±0.538 | 0.145 ±0.498 | 0.000[0.000-0.007] | 0.000[0.000-0.001] | 0.013 | 0.022 | 0.552 | 0.945 |
| ***G. Butyrivibrio*** | 0.247 ±0.619 | 0.236 ±0.816 | 0.000[0.000-0.000] | 0.000[0.000-0.000] | -0.02 | 0.04 | 0.615 | 0.945 |
| ***G. Turicibacter*** | 0.111 ±0.211 | 0.264 ±0.534 | 0.000[0.000-0.123] | 0.020[0.000-0.187] | 0.111 | 0.038 | 0.003 | 0.132 |
| ***G. Succinivibrio*** | 0.001 ±0.008 | 0.006 ±0.019 | 0.000[0.000-0.000] | 0.000[0.000-0.000] | -0.008 | NA | NA | NA |
| ***G. Intestinimonas*** | 0.013 ±0.038 | 0.017 ±0.057 | 0.000[0.000-0.002] | 0.000[0.000-0.009] | -0.015 | 0.034 | 0.656 | 0.962 |
| ***G. Lachnospiraceae UCG-003*** | 0.019 ±0.042 | 0.027 ±0.125 | 0.000[0.000-0.014] | 0.000[0.000-0.000] | 0.003 | 0.008 | 0.702 | 0.976 |
| ***G. Muribaculaceae*** | 0.811 ±1.929 | 0.987 ±2.029 | 0.000[0.000-0.321] | 0.035[0.000-1.205] | 0.064 | 0.033 | 0.055 | 0.384 |
| ***G. Methanobrevibacter*** | 0.568 ±1.355 | 0.767 ±2.096 | 0.021[0.000-0.471] | 0.027[0.000-0.341] | -0.002 | 0.022 | 0.936 | 0.995 |
| ***G. Oscillospira*** | 0.046 ±0.182 | 0.040 ±0.171 | 0.000[0.000-0.021] | 0.000[0.000-0.013] | 0.011 | NA | NA | NA |
| ***G. Christensenella*** | 0.006 ±0.016 | 0.005 ±0.010 | 0.000[0.000-0.000] | 0.000[0.000-0.002] | -0.049 | 0.029 | 0.099 | 0.498 |
| ***G. Butyricimonas*** | 0.142 ±0.175 | 0.129 ±0.166 | 0.060[0.000-0.254] | 0.056[0.000-0.187] | 0.005 | 0.016 | 0.749 | 0.976 |
| ***G. Flavonifractor*** | 0.043 ±0.095 | 0.027 ±0.061 | 0.000[0.000-0.034] | 0.000[0.000-0.016] | -0.035 | 0.025 | 0.171 | 0.652 |
| ***G. Eubacterium xylanophilum group*** | 0.229 ±0.277 | 0.246 ±0.329 | 0.116[0.038-0.376] | 0.113[0.061-0.300] | 0.004 | 0.014 | 0.767 | 0.976 |
| ***G. Eubacterium siraeum group*** | 0.431 ±0.755 | 0.343 ±0.535 | 0.114[0.000-0.459] | 0.178[0.003-0.364] | 0.007 | 0.019 | 0.711 | 0.976 |
| ***G. Odoribacter*** | 0.586 ±1.014 | 0.473 ±0.852 | 0.235[0.118-0.568] | 0.238[0.172-0.455] | -0.001 | 0.009 | 0.932 | 0.995 |
| ***F. Oscillospiraceae-003*** | 0.338 ±0.533 | 0.376 ±0.614 | 0.105[0.036-0.353] | 0.203[0.056-0.456] | 0.024 | 0.016 | 0.134 | 0.563 |
| ***G. Paraprevotella*** | 0.465 ±1.058 | 0.438 ±0.972 | 0.076[0.000-0.312] | 0.079[0.012-0.305] | -0.002 | 0.014 | 0.881 | 0.995 |
| ***G. Alistipes*** | 4.896 ±4.099 | 5.718 ±4.908 | 4.241[2.457-6.255] | 4.802[2.585-6.964] | 0.013 | 0.01 | 0.178 | 0.659 |
| ***G. Lachnospiraceae NK4A136 group*** | 1.350 ±1.189 | 1.411 ±1.436 | 0.996[0.389-1.919] | 1.071[0.496-1.815] | 0.005 | 0.01 | 0.598 | 0.945 |
| ***F. Oscillospiraceae-002*** | 2.611 ±2.672 | 3.099 ±2.779 | 2.157[0.820-3.392] | 2.202[1.119-3.997] | 0.022 | 0.011 | 0.05 | 0.375 |
| ***G. Barnesiella*** | 1.282 ±1.251 | 1.217 ±1.282 | 0.978[0.326-1.939] | 0.805[0.358-1.813] | 0.006 | 0.015 | 0.685 | 0.976 |
| ***G. Asteroleplasma*** | 0.017 ±0.113 | 0.008 ±0.040 | 0.000[0.000-0.000] | 0.000[0.000-0.000] | 0.061 | NA | NA | NA |
| ***G. Rikenella*** | 0.004 ±0.008 | 0.003 ±0.008 | 0.000[0.000-0.006] | 0.000[0.000-0.000] | 0.012 | 0.023 | 0.618 | 0.945 |
| ***F. Oscillospiraceae-005*** | 0.636 ±0.626 | 0.651 ±0.686 | 0.471[0.179-0.907] | 0.515[0.157-0.815] | 0.001 | 0.012 | 0.901 | 0.995 |
| ***O. Coriobacteriales g1*** | 0.104 ±0.199 | 0.101 ±0.231 | 0.012[0.000-0.111] | 0.009[0.000-0.055] | -0.013 | 0.015 | 0.398 | 0.851 |
| ***G. Allisonella*** | 0.022 ±0.089 | 0.036 ±0.187 | 0.000[0.000-0.000] | 0.000[0.000-0.000] | 0.042 | NA | NA | NA |
| ***G. Blautia*** | 5.000 ±4.741 | 6.310 ±7.453 | 3.778[2.081-6.375] | 3.976[1.876-6.867] | 0.01 | 0.011 | 0.373 | 0.84 |
| ***G. Dorea*** | 1.388 ±1.159 | 1.289 ±1.063 | 1.064[0.525-1.829] | 1.085[0.522-1.793] | -0.007 | 0.009 | 0.488 | 0.945 |
| ***F. Lachnospiraceae-900066575*** | 0.021 ±0.030 | 0.016 ±0.023 | 0.004[0.000-0.031] | 0.006[0.000-0.021] | -0.037 | 0.015 | 0.012 | 0.234 |
| ***G. Erysipelotrichaceae UCG-003*** | 0.668 ±1.115 | 0.723 ±1.148 | 0.211[0.105-0.704] | 0.348[0.147-0.762] | 0.024 | 0.013 | 0.062 | 0.41 |
| ***G. Romboutsia*** | 0.496 ±0.769 | 0.472 ±0.994 | 0.167[0.053-0.584] | 0.146[0.029-0.427] | -0.031 | 0.019 | 0.107 | 0.498 |
| ***G. Escherichia-Shigella*** | 1.199 ±4.124 | 1.166 ±4.111 | 0.031[0.000-0.165] | 0.033[0.004-0.125] | -0.017 | NA | NA | NA |
| ***G. Lactobacillus*** | 0.285 ±1.392 | 0.116 ±0.465 | 0.013[0.000-0.058] | 0.005[0.000-0.030] | 0.032 | NA | NA | NA |
| ***F. Butyricicoccaceae-009*** | 0.018 ±0.044 | 0.013 ±0.022 | 0.000[0.000-0.022] | 0.000[0.000-0.023] | -0.016 | 0.018 | 0.379 | 0.84 |
| ***G. Ruminococcus gauvreauii group*** | 0.227 ±0.364 | 0.212 ±0.378 | 0.121[0.014-0.268] | 0.085[0.000-0.177] | -0.026 | 0.015 | 0.085 | 0.454 |
| ***G. Ruminococcus torques group*** | 0.587 ±0.724 | 0.574 ±0.766 | 0.388[0.131-0.599] | 0.322[0.132-0.679] | 0.002 | 0.013 | 0.871 | 0.995 |
| ***F. Christensenellaceae*** | 0.020 ±0.036 | 0.015 ±0.023 | 0.005[0.000-0.030] | 0.007[0.000-0.026] | -0.016 | 0.018 | 0.38 | 0.84 |
| ***G. Enterorhabdus*** | 0.064 ±0.128 | 0.044 ±0.110 | 0.000[0.000-0.068] | 0.000[0.000-0.049] | -0.049 | 0.027 | 0.07 | 0.41 |
| ***F. Eggerthellaceae*** | 0.242 ±0.377 | 0.163 ±0.209 | 0.036[0.000-0.394] | 0.054[0.000-0.292] | -0.037 | 0.016 | 0.026 | 0.303 |
| ***G. Adlercreutzia*** | 0.116 ±0.257 | 0.105 ±0.235 | 0.000[0.000-0.067] | 0.001[0.000-0.071] | -0.003 | 0.012 | 0.789 | 0.982 |
| ***G. Ruminococcus gnavus group*** | 0.077 ±0.263 | 0.024 ±0.076 | 0.000[0.000-0.016] | 0.000[0.000-0.000] | -0.037 | 0.024 | 0.116 | 0.521 |
| ***G. Collinsella*** | 0.441 ±0.861 | 0.330 ±0.485 | 0.198[0.000-0.430] | 0.138[0.000-0.518] | -0.016 | 0.016 | 0.319 | 0.825 |
| ***F. Prevotellaceae*** | 0.203 ±0.376 | 0.179 ±0.344 | 0.000[0.000-0.225] | 0.000[0.000-0.205] | -0.021 | 0.021 | 0.316 | 0.825 |
| ***G. Catenibacterium*** | 0.229 ±0.881 | 0.251 ±0.700 | 0.000[0.000-0.011] | 0.000[0.000-0.004] | < 0.001 | 0.023 | 0.992 | 0.999 |
| ***P. Firmicutes-DTU014*** | 0.017 ±0.030 | 0.014 ±0.025 | 0.000[0.000-0.024] | 0.000[0.000-0.017] | -0.02 | 0.023 | 0.372 | 0.84 |
| ***G. Eisenbergiella*** | 0.023 ±0.075 | 0.078 ±0.450 | 0.000[0.000-0.005] | 0.000[0.000-0.007] | 0.04 | NA | NA | NA |
| ***G. Olsenella*** | 0.012 ±0.032 | 0.013 ±0.029 | 0.000[0.000-0.000] | 0.000[0.000-0.002] | 0.004 | 0.021 | 0.842 | 0.982 |
| ***G. Lactococcus*** | 0.017 ±0.038 | 0.007 ±0.023 | 0.000[0.000-0.019] | 0.000[0.000-0.000] | -0.003 | 0.042 | 0.934 | 0.995 |

Intervention effects are indicated by the model coefficient and standard error of MaAsLin2 statistical models. Model coefficients represent the effect size, positive coefficients indicate an increased relative abundance of a specific genus. Only significant, diet-induced changes with FDR adjusted p-value (q-values) equal or less than 0.0.5 in one or more phenotype x diet combinations being statistically significant. FDR, false discovery rate; HMUFA, high mono-unsaturated fatty acid diet; LFHP, low fat, high protein, high fiber diet; LIR, liver insulin resistance; MIR, muscle insulin resistance.

**Supplementary Table 10. Changes in individual fecal microbial genus abundances after a 12-week HMUFA or LFHP diet, related to Figure 3. Prevalence cutoff >25%.**

| **Taxa** | **Mean ±SD(%)** | **Mean ±SD(%)** | **Median[IQR:25%-75%](%)** | **Median[IQR:25%-75%](%)** | **Coef** | **SE** | **P-value** | **Q-value** |
| --- | --- | --- | --- | --- | --- | --- | --- | --- |
|  | **{Week 0}** | **{Week 12}** | **{Week 0}** | **{Week 12}** |  |  |  |  |
| **HMUFA** | | | | | | | | |
| ***G. Prevotellaceae NK3B31 group*** | 0.250 ±1.001 | 0.522 ±2.248 | 0.000[0.000-0.000] | 0.000[0.000-0.000] | 0.218 | 0.012 | < 0.001 | < 0.001 |
| ***G. Turicibacter*** | 0.115 ±0.227 | 0.111 ±0.216 | 0.009[0.000-0.079] | 0.025[0.000-0.089] | < 0.001 | 0.017 | 0.978 | 0.978 |
| ***G. Succinivibrio*** | 0.017 ±0.055 | 0.064 ±0.467 | 0.000[0.000-0.007] | 0.000[0.000-0.010] | 0.059 | 0.015 | < 0.001 | 0.001 |
| ***G. Muribaculaceae*** | 0.818 ±2.395 | 1.040 ±2.577 | 0.000[0.000-0.476] | 0.006[0.000-0.857] | 0.054 | 0.015 | < 0.001 | 0.004 |
| ***G. Dialister*** | 1.012 ±2.266 | 0.990 ±2.152 | 0.025[0.000-1.087] | 0.016[0.000-1.168] | 0.008 | 0.011 | 0.464 | 0.743 |
| ***G. Lactobacillus*** | 0.332 ±1.415 | 0.041 ±0.131 | 0.005[0.000-0.066] | 0.000[0.000-0.015] | 0.069 | NA | NA | NA |
| ***G. Sutterella*** | 0.377 ±0.713 | 0.433 ±0.871 | 0.162[0.042-0.325] | 0.183[0.063-0.461] | 0.032 | 0.011 | 0.005 | 0.036 |
| ***F. Oscillospiraceae-003*** | 0.336 ±0.403 | 0.476 ±0.528 | 0.207[0.073-0.486] | 0.338[0.122-0.692] | 0.032 | 0.009 | < 0.001 | 0.005 |
| ***G. Eubacterium eligens group*** | 0.298 ±0.673 | 0.351 ±0.538 | 0.124[0.040-0.280] | 0.156[0.049-0.448] | 0.025 | 0.01 | 0.011 | 0.068 |
| ***G. Lachnospiraceae NK4A136 group*** | 1.615 ±1.828 | 2.063 ±1.954 | 1.091[0.546-2.157] | 1.444[0.706-2.825] | 0.023 | 0.007 | 0.001 | 0.013 |
| ***G. Clostridia vadinBB60 group*** | 0.122 ±0.315 | 0.107 ±0.171 | 0.028[0.004-0.137] | 0.031[0.005-0.121] | 0.023 | 0.003 | < 0.001 | < 0.001 |
| ***G. Coprococcus*** | 1.957 ±1.648 | 1.602 ±1.412 | 1.403[0.811-2.614] | 1.064[0.521-2.237] | -0.02 | 0.007 | 0.005 | 0.038 |
| ***G. Blautia*** | 5.106 ±4.140 | 4.159 ±3.953 | 3.690[2.277-6.525] | 2.913[1.483-5.569] | -0.02 | 0.006 | 0.002 | 0.016 |
| ***G. Anaerostipes*** | 1.877 ±2.847 | 1.542 ±2.650 | 1.057[0.376-2.322] | 0.569[0.253-2.007] | -0.023 | 0.009 | 0.012 | 0.071 |
| ***G. Ruminococcus gauvreauii group*** | 0.267 ±0.344 | 0.188 ±0.253 | 0.146[0.061-0.343] | 0.119[0.012-0.233] | -0.024 | 0.009 | 0.01 | 0.068 |
| ***G. Dorea*** | 1.417 ±1.132 | 1.140 ±1.204 | 1.174[0.677-1.683] | 0.757[0.382-1.544] | -0.025 | 0.008 | 0.001 | 0.008 |
| ***F. Butyricicoccaceae-009*** | 0.018 ±0.036 | 0.013 ±0.022 | 0.003[0.000-0.020] | 0.005[0.000-0.017] | -0.033 | 0.013 | 0.01 | 0.068 |
| ***G. Bifidobacterium*** | 1.110 ±1.464 | 0.877 ±1.312 | 0.601[0.178-1.262] | 0.319[0.077-1.231] | -0.023 | 0.011 | 0.042 | 0.141 |
| ***F. Lachnospiraceae-900066575*** | 0.023 ±0.030 | 0.021 ±0.026 | 0.014[0.000-0.037] | 0.017[0.000-0.032] | -0.006 | 0.009 | 0.524 | 0.774 |
| ***G. Senegalimassilia*** | 0.117 ±0.446 | 0.090 ±0.352 | 0.016[0.000-0.094] | 0.000[0.000-0.049] | -0.035 | 0.012 | 0.003 | 0.028 |
| ***G. Erysipelotrichaceae UCG-003*** | 0.795 ±1.053 | 0.535 ±0.655 | 0.444[0.135-0.930] | 0.303[0.071-0.675] | -0.036 | 0.009 | < 0.001 | 0.002 |
| ***G. Holdemanella*** | 1.119 ±2.237 | 0.703 ±1.417 | 0.031[0.000-0.925] | 0.022[0.000-0.758] | -0.035 | 0.016 | 0.032 | 0.127 |
| ***F. Eggerthellaceae*** | 0.271 ±0.503 | 0.166 ±0.316 | 0.060[0.000-0.340] | 0.033[0.000-0.184] | -0.039 | 0.012 | 0.001 | 0.008 |
| ***G. Candidatus Soleaferrea*** | 0.009 ±0.019 | 0.005 ±0.012 | 0.002[0.000-0.010] | 0.000[0.000-0.006] | -0.04 | 0.011 | < 0.001 | 0.004 |
| ***G. Ruminococcus torques group*** | 0.739 ±1.062 | 0.429 ±0.528 | 0.453[0.192-0.954] | 0.265[0.117-0.531] | -0.041 | 0.008 | < 0.001 | < 0.001 |
| ***G. Lachnospiraceae UCG-004*** | 0.036 ±0.117 | 0.026 ±0.054 | 0.000[0.000-0.013] | 0.000[0.000-0.030] | -0.042 | 0.017 | 0.011 | 0.068 |
| ***G. Collinsella*** | 0.448 ±0.556 | 0.286 ±0.433 | 0.271[0.061-0.662] | 0.139[0.057-0.315] | -0.045 | 0.008 | < 0.001 | < 0.001 |
| ***G. Adlercreutzia*** | 0.127 ±0.268 | 0.088 ±0.170 | 0.000[0.000-0.125] | 0.007[0.000-0.083] | -0.046 | 0.015 | 0.002 | 0.019 |
| ***P. Firmicutes-DTU014*** | 0.023 ±0.052 | 0.024 ±0.047 | 0.000[0.000-0.022] | 0.000[0.000-0.031] | 0.005 | 0.014 | 0.726 | 0.887 |
| ***G. Lactococcus*** | 0.109 ±0.555 | 0.002 ±0.006 | 0.000[0.000-0.024] | 0.000[0.000-0.000] | -0.262 | 0.046 | < 0.001 | < 0.001 |
| **LFHP** | | | | | | | | |
| ***G. Prevotellaceae NK3B31 group*** | 0.427 ±1.793 | 0.519 ±2.117 | 0.000[0.000-0.000] | 0.000[0.000-0.001] | 0.006 | 0.024 | 0.806 | 0.919 |
| ***G. Turicibacter*** | 0.097 ±0.192 | 0.257 ±0.498 | 0.002[0.000-0.071] | 0.015[0.000-0.191] | 0.089 | 0.022 | < 0.001 | 0.002 |
| ***G. Succinivibrio*** | 0.028 ±0.208 | 0.025 ±0.181 | 0.000[0.000-0.000] | 0.000[0.000-0.000] | 0.05 | 0.012 | < 0.001 | 0.001 |
| ***G. Muribaculaceae*** | 0.788 ±1.781 | 0.964 ±1.920 | 0.004[0.000-0.531] | 0.025[0.000-1.084] | 0.032 | 0.022 | 0.152 | 0.506 |
| ***G. Dialister*** | 1.030 ±1.838 | 1.455 ±2.485 | 0.032[0.000-1.170] | 0.068[0.000-1.990] | 0.041 | 0.015 | 0.005 | 0.084 |
| ***G. Lactobacillus*** | 0.282 ±1.355 | 0.120 ±0.456 | 0.011[0.000-0.057] | 0.003[0.000-0.030] | 0.036 | 0.006 | < 0.001 | < 0.001 |
| ***G. Sutterella*** | 0.292 ±0.402 | 0.241 ±0.344 | 0.122[0.038-0.362] | 0.098[0.018-0.338] | -0.014 | 0.013 | 0.28 | 0.714 |
| ***F. Oscillospiraceae-003*** | 0.312 ±0.451 | 0.400 ±0.601 | 0.104[0.038-0.388] | 0.187[0.057-0.542] | 0.029 | 0.013 | 0.026 | 0.26 |
| ***G. Eubacterium eligens group*** | 0.227 ±0.352 | 0.252 ±0.345 | 0.081[0.024-0.274] | 0.134[0.051-0.299] | 0.018 | 0.012 | 0.146 | 0.5 |
| ***G. Lachnospiraceae NK4A136 group*** | 1.363 ±1.124 | 1.476 ±1.753 | 1.102[0.472-1.893] | 1.012[0.506-1.850] | 0.005 | 0.008 | 0.516 | 0.878 |
| ***G. Clostridia vadinBB60 group*** | 0.222 ±0.991 | 0.178 ±0.629 | 0.027[0.000-0.123] | 0.026[0.001-0.123] | -0.001 | 0.018 | 0.969 | 0.984 |
| ***G. Coprococcus*** | 1.902 ±1.593 | 1.917 ±1.517 | 1.323[0.739-2.733] | 1.556[0.639-3.093] | 0.002 | 0.007 | 0.717 | 0.898 |
| ***G. Blautia*** | 5.620 ±4.900 | 6.104 ±6.385 | 4.119[2.250-7.341] | 4.013[1.974-7.782] | 0.005 | 0.007 | 0.508 | 0.878 |
| ***G. Anaerostipes*** | 1.952 ±2.445 | 1.791 ±3.441 | 0.986[0.456-2.229] | 0.911[0.280-1.858] | -0.012 | 0.009 | 0.205 | 0.606 |
| ***G. Ruminococcus gauvreauii group*** | 0.215 ±0.310 | 0.204 ±0.329 | 0.118[0.009-0.281] | 0.089[0.000-0.256] | -0.01 | 0.01 | 0.317 | 0.743 |
| ***G. Dorea*** | 1.343 ±1.091 | 1.293 ±1.038 | 1.053[0.538-1.661] | 1.070[0.561-1.722] | 0.002 | 0.007 | 0.754 | 0.898 |
| ***F. Butyricicoccaceae-009*** | 0.023 ±0.060 | 0.044 ±0.271 | 0.000[0.000-0.023] | 0.000[0.000-0.022] | 0.008 | 0.013 | 0.563 | 0.898 |
| ***G. Bifidobacterium*** | 1.071 ±1.175 | 0.717 ±0.866 | 0.725[0.260-1.433] | 0.401[0.110-1.050] | -0.034 | 0.011 | 0.002 | 0.048 |
| ***F. Lachnospiraceae-900066575*** | 0.022 ±0.027 | 0.016 ±0.020 | 0.015[0.000-0.038] | 0.011[0.000-0.022] | -0.035 | 0.009 | < 0.001 | 0.008 |
| ***G. Senegalimassilia*** | 0.069 ±0.131 | 0.047 ±0.076 | 0.018[0.000-0.102] | 0.005[0.000-0.060] | -0.022 | 0.011 | 0.052 | 0.374 |
| ***G. Erysipelotrichaceae UCG-003*** | 0.665 ±1.077 | 0.717 ±1.145 | 0.239[0.104-0.747] | 0.335[0.141-0.688] | 0.018 | 0.01 | 0.073 | 0.419 |
| ***G. Holdemanella*** | 1.194 ±1.913 | 0.931 ±1.939 | 0.234[0.000-1.658] | 0.102[0.000-0.928] | -0.039 | 0.014 | 0.005 | 0.084 |
| ***F. Eggerthellaceae*** | 0.260 ±0.494 | 0.235 ±0.657 | 0.043[0.000-0.334] | 0.058[0.000-0.228] | -0.023 | 0.014 | 0.109 | 0.455 |
| ***G. Candidatus Soleaferrea*** | 0.007 ±0.013 | 0.005 ±0.008 | 0.003[0.000-0.009] | 0.001[0.000-0.006] | 0.027 | NA | NA | NA |
| ***G. Ruminococcus torques group*** | 0.676 ±0.743 | 0.571 ±0.662 | 0.487[0.170-0.829] | 0.373[0.171-0.752] | -0.005 | 0.01 | 0.585 | 0.898 |
| ***G. Lachnospiraceae UCG-004*** | 0.020 ±0.060 | 0.027 ±0.078 | 0.000[0.000-0.015] | 0.000[0.000-0.023] | 0.005 | NA | NA | NA |
| ***G. Collinsella*** | 0.477 ±0.730 | 0.392 ±0.489 | 0.283[0.103-0.529] | 0.237[0.066-0.519] | -0.011 | 0.01 | 0.273 | 0.711 |
| ***G. Adlercreutzia*** | 0.095 ±0.215 | 0.083 ±0.196 | 0.000[0.000-0.068] | 0.000[0.000-0.052] | -0.02 | 0.014 | 0.141 | 0.5 |
| ***P. Firmicutes-DTU014*** | 0.017 ±0.030 | 0.012 ±0.024 | 0.000[0.000-0.024] | 0.000[0.000-0.013] | -0.05 | 0.015 | 0.001 | 0.03 |
| ***G. Lactococcus*** | 0.016 ±0.034 | 0.008 ±0.022 | 0.000[0.000-0.016] | 0.000[0.000-0.000] | -0.013 | 0.027 | 0.626 | 0.898 |

Intervention effects are indicated by the model coefficient and standard error of MaAsLin2 statistical models. Model coefficients represent the effect size, positive coefficients indicate an increased relative abundance of a specific genus. Only significant, diet-induced changes with FDR adjusted p-value (q-values) equal or less than 0.05 considered statistically different. ASV, amplicon sequence variant; FDR, false discovery rate; HMUFA, high mono-unsaturated fatty acid diet; LFHP, low fat, high protein, high fiber diet.

**Supplementary Table 11. Spearman associations between changes in cardiometabolic outcome parameters between week 0 and 12 and genera abundance at week 0 (baseline) or differences in taxa abundance between week 0 and week 12 (Shift).**

| **Taxa** | **Outcome** | **Estimate** | **P-value** | **Diet-Phenotype** |
| --- | --- | --- | --- | --- |
| **Baseline** | | | | |
| *G. Barnesiella* | MISI | 0.45017 | 0.00064 | HMUFA-MIR |
| *G. Sutterella* | CRP | 0.5663 | 0.00011 | HMUFA-LIR |
| *O. Rhodospirillales* | HOMA_IR | -0.57822 | 0.00008 | HMUFA-LIR |
| **Shift** | | | | |
| *F. Ruminococcaceae g1* | MISI | -0.44303 | 0.00079 | HMUFA-MIR |
| *F. Ruminococcaceae_DTU089* | MISI | -0.47209 | 0.00031 | HMUFA-MIR |
| *G. Oscillibacter* | MISI | 0.55081 | 0.00028 | HMUFA-LIR |

The table contains correlations between selected cardiometabolic outcome parameters and microbial genera abundance considered important (p-values ≤ 0.001 and coefficient ≥ 0.4). Data indicate whether the analysis was based on genera abundance values at baseline or the change between week 12 and baseline (Shift). CRP, C-reactive protein; HMUFA, high mono-unsaturated fatty acids; HOMA-IR, Homeostatic Model Assessment for Insulin Resistance; LFHP, low-fat high-protein high fiber; LIR, liver insulin resistance; MIR, muscle insulin resistance; MISI, muscle insulin sensitivity index.
